# A Tale of Two Tweets: What Factors Predict Forgiveness of Past Transgressions on Social Media?

**DOI:** 10.1177/01461672231214629

**Published:** 2023-12-12

**Authors:** Andrew J. Dawson, Sarah Williams, Anne E. Wilson

**Affiliations:** 1 Wilfrid Laurier University, Waterloo, Ontario, Canada

**Keywords:** moral judgment, politics, social media, cancel culture, racism

## Abstract

As more of our lives take place online, it is increasingly common for public figures to have their current image tarnished by their mistakes and transgressions in what is often the distant past. Three experiments (*N* = 2,296) found that judgments of a public figure who tweeted racist statements in the past were less harsh when more time had passed and when the public figure was younger at the time of the tweet. However, politics also played a powerful role. Independent of time and age, liberals allowed less possibility of redemption for anti-Black tweets, while conservatives were less forgiving for anti-White tweets. Such partisan differences extended not only to moral judgments of the individual, but also general moral principles and participants’ subjective perceptions of the situation itself, including subjective temporal distance from the tweet, the subjective age of the public figure, and the current relevance of the past statements.

*“It's one of the greatest gifts you can give yourself: to forgive. Forgive everybody.”*

*- Maya Angelou*

*“When someone shows you who they are, believe them the first time.”*

*- Maya Angelou*

The temporal landscape of every life contains multitudes, and virtually everyone has a history of both commendable and condemnable acts. People may look back on their own past regrettable choices and hope that these moments don’t define them forever in the eyes of others. Likewise, people must weigh the recent and remote acts of others to decide on what defines them in the present. Does a past harm reflect something about the offender’s current character? Have they grown, changed, or made amends? Have the consequences of the harm dissipated or do the wounds remain raw? Does the past misdeed likely foretell future transgressions? Observers can take numerous factors into account in judging a person’s past deeds, including the type and severity of transgression committed, the circumstances of the offense, the perceived intent of the transgressor, and time that has passed since the transgression and their beliefs about the possibility of moral change (Adams et al., 2022; Leith et al., 2014; McCullough et al., 2010; Ward & Wilson, 2015; Wohl & McGrath, 2007; Young & Saxe, 2011). In essence, the perceiver’s moral calculus determines the balance between condemnation and redemption; whether to hold onto the past act as presently pertinent, or to forgive and forget.

When these moral judgments occur within personal relationships, they are often made within the context of rich knowledge of the person’s usual behavior and their evolution across time, and can shape decisions about relationship maintenance or exit and inform who to trust and distrust in broader interpersonal communities. These judgments may not be perfectly calibrated but may serve as a reasonably functional way to navigate social life and maintain social order in communities.

In contrast, people are also often faced with judgments to make about distant acquaintances, strangers, and public figures on the basis of considerably less information. When faced with only a selected snapshot of a stranger or public figure’s past, it can be difficult to know how to weigh this evidence plucked from some moment in the often-distant past.

Indeed, the past does not always stay in the past once it has become part of the permanent record of social media. Countless stories have emerged in recent years in which the past statements or actions of public figures have resurfaced to tarnish their character in the present. In many cases, these transgressions occurred quite long ago, evoking little reaction at the time. Nonetheless, upon rediscovery (and wide circulation online), they evoked much moral condemnation and calls for repercussions. Prominent cases include Kevin Hart, who lost his chance to host the Oscars after long-past homophobic jokes resurfaced (Daw, 2020), Alexi McCammond, who stepped down from her appointment as Editor-in-chief at Teen Vogue when anti-Asian and homophobic tweets from her teen years began circulating (Robertson, 2021), and writer-director James Gunn, who was fired from Guardians of the Galaxy when old tweets joking about pedophilia and sexual assault resurfaced (McArdle, 2018). Specific examples noted in this article will quickly become outdated and replaced with new incidents. What is important is not the individual examples themselves, but the larger pattern they illustrate. What once may have been buried in the past is now preserved digitally in perpetuity and can emerge years later to public outcry and widespread dissemination on social media.

Some view the backlash in such cases as the just consequences of one’s actions (Hagi, 2019; Tensley, 2020); the term “accountability culture” reflects the idea that the democratization of speech on social media has simply resulted in bad actors finally being held responsible for their moral misdeeds. Others view it as censorship and mob rule in which the punishment far outstrips the crime (Furdyk, 2020; Romano, 2020), reflected in the often-used term “cancel culture.” Given the quantity and variety of such cases, however, a single analysis or narrative will not always apply; there are surely both cases where online accountability served a crucial justice function, and other cases where it results in miscalibrated sanctions. We suggest that, regardless of individuals’ normative stance on what *should* occur in such cases, this new digital context for moral approbation is important to understand, psychologically and societally. We argue that it is important to study how people weigh moral judgment of online public figures because the digital environment is playing an increasingly large role in social interaction, judgment, and social learning about morality. Although recent research has begun to untangle some of these online moral dynamics (Brady & Van Bavel, 2021; Crockett, 2017; Dias et al., 2022; Sawaoka & Monin, 2018; Schumann, 2018; Schumann & Dragotta, 2020), many questions remain. In the current research, we focused particularly on one type of moral offense that may become increasingly relevant in our new digital environment: The sometimes long-past transgression that resurfaces in the present. As the title of Jeffrey Rosen’s prescient 2010 article highlights, “the web means the end of forgetting.” He reflected on how modern society had only begun to grapple with how the permanence of the internet’s public record could flatten the temporal landscape of people’s lives, “. . .threatening, at an almost existential level, our ability to control our identities; to preserve the option of reinventing ourselves and starting anew; to overcome our checkered pasts” (Rosen, 2010).

## Factors Affecting Forgiveness of Past Transgressions

### Objective Time and Age

There are a number of factors that influence one’s willingness to forgive, including the severity of the transgression (Fincham et al., 2005), the perceived intent of the transgressor (Young & Saxe, 2011), and the quality of the apology (Schumann, 2018; Schumann & Dragotta, 2020), but we focus on factors with particular relevance for current judgment of transgressions that occurred some distance in the past. First, we know that the passage of time is linked to increased forgiveness for past transgressions (McCullough et al., 2010; Wohl & McGrath, 2007). Second, when we judge a person for a long-past transgression, we are judging their considerably younger self. The age of the transgressor is relevant to moral judgment because the capacity for decision-making and impulse control are domains still under considerable development until adulthood, explaining adolescents’ greater inclination toward risky decisions and incomplete understanding of the impact of their actions adolescents (Blakemore, 2008; Blakemore & Robbins, 2012; Casey et al., 2011; Mallya et al., 2019; Selemon, 2013; Steinberg, 2013). For this reason, adolescents may not be considered as fully responsible moral agents in the same way as adults. To our knowledge, relatively little psychological research has been done to examine how the age of a moral transgressor influences the judgments of perceivers. Nonetheless, adolescents are recognized as not fully responsible agents in many societies’ legal systems, recommending lighter punishments for equivalent crimes (“The youth criminal justice act summary and background,” 2021; “Youth in the justice system: An overview,” 2019). The Juvenile Law Center website explicitly states that “. . .children who commit crimes are different from adults; as a class, they are less blameworthy, and they have a greater capacity for change” (“Youth in the justice system: An overview,” 2019, para. 1).

### Subjective Time and Age

Although we argue that the chronological passage of time and actual age of the offender may be taken into account in moral judgment, there is reason to expect that people’s psychological experience of these dimensions may depart from their objective states. Subjective time refers to how long it *feels* it has been since a given event from the perspective of the observer (Ross & Wilson, 2002). Although subjective time may be related to objective time, these two judgments can diverge in systematic ways. For instance, people tend to feel subjectively closer to equidistant desirable than undesirable personal past events in their own lives and people in satisfied relationships relegate their partners’ transgressions to the subjectively distant past while unhappy or insecurely attached partners keep viewing past harms as psychologically recent (Cortes & Wilson, 2016; Cortes et al., 2018; Wilson et al., 2009). Subjectively distancing past harms can make them feel less relevant, and hence less threatening, to the present (Broemer et al., 2008; Cortes et al., 2018; Van Boven & Caruso, 2015), whereas keeping past misdeeds close in time may keep them feeling more psychologically pertinent to present judgments. People tend to shift the subjective distance of past events when it helps them reach desired conclusions about themselves or others in the present (Dawson et al., 2022; Wilson et al., 2009). Therefore, in the current studies, we expected that people may attend to the actual passage of time but also to their *subjective* sense of whether the offense was long ago or still quite recent.

Similar to subjective time, subjective age is how young or old a person subjectively seems to be, independent of actual age. Although some work has examined people’s subjective perception of their own age (e.g., Rubin & Berntsen, 2006), the literature on subjective perception of others’ age is relatively limited. However, research on racial bias in the justice system sheds some light on the issue, revealing that Black juvenile offenders are seen as subjectively older than white ones (Goff et al., 2014; Rattan et al., 2012), which may underlie their harsher treatment for similar offenses. This may suggest that if people encounter online misbehavior done by a much younger version of a public figure, their *subjective* view of that person’s age will matter as well as their actual age at the time of the offense.

## Politics

### Political Polarization and Motivated Cognition

Although people may claim that they are principled about their moral judgment, evidence suggests that people often reason about moral problems in a directional manner (motivated reasoning) that aligns with prior beliefs or allegiances (Ditto et al., 2009; Uhlmann et al., 2009). Political affiliation may be an increasingly salient factor in people’s motivated moral reasoning. As political polarization and animosity continue to rise (Abramowitz & Webster, 2018; Iyengar et al., 2012), the temptation to exacerbate or minimize moral fault depending on how it aligns with one’s partisan sensibilities may increase accordingly. This may lead people with different political identities to condemn some acts more harshly than others depending on the degree to which those infractions offend their ingroup’s worldviews or values (Dias et al., 2022; Smith, Ratliff et al., 2019; Tetlock, 2003). In the online sphere, the likelihood of outrage and calls for “canceling” often sort across party lines, with both sides targeting the other for perceived infractions (Bishop, 2018; Bradley, 2018). All of this is likely exacerbated by the outrage-amplifying features of the online ecosystem (Brady et al., 2020; Crockett, 2017).

Because people are often more motivated to protect their cherished ingroup beliefs and identities than they are motivated to be accurate (Kahan et al., 2017; Kunda, 1990), the process of politically motivated moral reasoning is directional, tipping the scale toward the desired conclusion (Chen et al., 1999; Erisen et al., 2014; Taber & Lodge, 2006). One way that people may build a psychological case for their preferred moral conclusion is by appealing to different moral principles depending on what principle justifies the conclusion they favor (Ditto et al., 2009; Uhlmann et al., 2009). For example, conservatives and liberals may shift their endorsement of principles regarding the sanctity of life vs. personal freedom depending on whether the topic is abortion or COVID-19 vaccination.

We build on past insights about motivated appeal to moral principles (also called political casuistry, Knowles & Ditto, 2012) by, first, broadening the category of moral principles to include potential *mitigating circumstances* that people may consider in making their moral judgments, such as the passage of time and age of offender. People may consider these factors as intuitive moral principles that inform when condemnation versus redemption should be possible. However, the subjective perception of time and age are malleable and are sometimes shifted in ways that support a desired conclusion about the self or others (Cortes et al., 2018; Goff et al., 2014; Peetz et al., 2010; Rattan et al., 2012; Rubin & Berntsen, 2006; Rudolph et al., 2019; Wilson et al., 2009). In line with the motivated use of moral principles, we reasoned that people may shift their perceptions of the subjective time and age of the offense, with implications for how relevant the transgression is to judgment in the present, which in turn could amplify or attenuate moral condemnation and subsequent punishment.

### Type of Offense

The current studies focus on a particular kind of moral offense: the expression of racist views. We began with the assumption that although most Americans reject racial discrimination in principle, political liberals perceive anti-Black racism to be a more pervasive problem and would have particular antipathy for expressions of anti-Black prejudice (Earle & Hodson, 2020; Norton & Sommers, 2011; Tetlock et al., 2000). However, we also sought to identify a type of moral infraction that political conservatives may find more offensive, and reasoned that perceptions of anti-White prejudice could provide a parallel test. Although people across the ideological divide acknowledge that Black people face discrimination, this belief is more strongly held by Democrats, whereas Republicans perceive more anti-White discrimination than Democrats (Earle & Hodson, 2020; Sheffield, 2019). Given these diverging perspectives on the prevalence of different kinds of discrimination, we expected that liberals may be more motivated to condemn cases of anti-Black racism while conservatives may be more motivated to condemn cases of anti-White racism.

## The Current Studies

The current research examined several potential factors that may be relevant to judgments of past public statements, in particular a series of racist tweets made by a public figure in the past. We aimed to investigate the causal effect of time and age. We also measured participants’ political leanings, and manipulated the race targeted by the derogatory statements to be either Black people or White people.

We expected that judgments would be more forgiving when more time had passed and the public figure was younger when they posted the tweets. We also expected liberals to give harsher judgments toward anti-Black tweets and conservatives’ harsher judgments toward anti-White tweets. Finally, we expected that people’s subjective perceptions of the circumstances (i.e., passage of time, and age of transgressor) would also shift in ways that reflect better or more poorly on the public figure depending on the observer’s politics. A full list of our formal hypotheses can be found in Table 1.

**Table 1:** Formal Hypotheses Preregistered for Each Study.

Hypothesis	Preregistered?		
	Study 1	Study 2	Study 3
Manipulation Check 1. Subjective temporal distance from the tweets will be shorter when they occurred 2 years ago rather than 7.	✕	✕	✓
Manipulation Check 2. Subjective age for the public figure will be younger when he was 16 rather than 28.	✕	✕	✓
Hypothesis 1a. Participants will judge the public figure more harshly for the tweets when they occurred 2 years ago rather than 7.	✕	✓	✓
Hypothesis 1b. Participants will judge the public figure more harshly for the tweets when he was 28 rather than 16.	✕	✓	✓
Hypothesis 2a. Liberal participants will judge the public figure more harshly for anti-Black tweets compared with anti-White tweets (and control tweets, in Study 3).	✕	✓	✓
Hypothesis 2b. Conservative participants will judge the public figure more harshly for anti-White tweets compared with anti-Black tweets (and control tweets, in Study 3).	✕	✓	✕
Hypothesis 3a. Liberal participants will view the surrounding circumstances in a way that places more blame on the public figure in the case of anti-Black tweets compared with anti-White tweets (and control tweets, in Study 3).	✕	✕	✓
Hypothesis 3b. Conservative participants will view the surrounding circumstances in a way that places more blame on the public figure in the case of anti-White tweets compared with anti-Black tweets (and control tweets, in Study 3).	✕	✕	✕
Hypothesis 4a. There will be a significant path model of moderated mediation, whereby the political leaning of the participants will interact with the race targeted by the tweets to predict subjective **time** from the tweets, which will predict judgment.	✕	✕	✓
Hypothesis 4b. There will be a significant path model of moderated mediation, whereby the political leaning of the participants will interact with the race targeted by the tweets to predict subjective **age** from the tweets, which will predict judgment.	✕	✕	✕
Hypothesis 5a. Liberal participants will endorse more judgmental and more punitive general principles regarding past offensive statements in the case of anti-Black tweets compared with anti-White tweets (and control tweets). [Study 3 Only]	N/A	N/A	✓
Hypothesis 5b. Conservative participants will endorse more judgmental and more punitive general principles regarding past offensive statements in the case of anti-White tweets compared with anti-Black tweets. (and control tweets) [Study 3 Only]	N/A	N/A	✕

*Note*. In Study 3, Hypotheses 2b, 3b, and 5b were actually preregistered, but they did not predict conservatives would respond more harshly to anti-White tweets, rather just that they would *not* respond more harshly to anti-Black tweets. This reflected our decreased confidence in the effect for conservatives following the null results in Study 2. The versions included in the table better reflect the patterns of interest, but we do not want to incorrectly claim that these versions were what we predicted in Study 3.

## Materials and Methods

Because all three studies use variations of the same method to test the same hypotheses, we report methods for all studies together then move to results for all three studies. For Studies 2 and 3, the design, sample selection, exclusion criteria, and planned analyses were preregistered. Preregistrations as well as study materials and data files can be found on OSF (https://osf.io/ag8w5/).

### Participants

The samples consisted of U.S. citizens 18 years old and above who self-selected into the studies on Mechanical Turk (Crowston, 2012; Study 1 Wave 1) and CloudResearch (formerly TurkPrime; Litman et al., 2017; Study 1 Wave 2, Studies 2 and 3) and completed a survey on Qualtrics (Qualtrics, 2018) in exchange for US$1.00 (Study 1) or US$2.00 (Studies 2 and 3) based on rates at the time. See Supporting Information for exact exclusions by condition for each study.

#### Study 1

In Study 1, which was exploratory and not preregistered, the data were collected in two separate waves, which reflected an evolving outlook on the research questions. The initial goal of the first study was to examine time and age, and as such only these two factors were originally manipulated. The racist statements in the first wave were exclusively anti-Black, so targeted race was not initially an experimental factor. Political differences in the first wave data prompted the collection of the second wave of data where all participants were exposed to anti-*White* tweets instead. Waves 1 and 2 were collected in December 2018 and March 2019, respectively, and were combined into a single sample (not including the Age 22 condition, see below) of 834 participants to allow comparison across tweet type, however, because race of tweet was not experimentally manipulated (and indeed, collected at different times), interpretations should be particularly cautious. Participants were excluded if they failed (or didn’t answer) one of two attention checks (79 participants), if they did not indicate at the end of the survey that they answered honestly (44 participants), if they displayed bot-like behavior such as clusters of responses from the same IP address (26 participants), or if they did not identify as leaning conservative or liberal (100 participants), which left a final sample of 634 (480 White, 64 Black; 438 liberal, 196 conservative; *M*_age_ = 37.31, *SD*_age_ = 11.49). According to G*Power (Faul et al., 2007), this sample gave us the power to detect an effect size of *f* = 0.11 (η_p_^2^ = 0.01) with 80% power and α = .05.

#### Study 2

To address the limited number of Black participants in Study 1, we recruited equal samples of Black and White participants in Study 2. In our original preregistered analyses, we planned to analyze time, age, and political orientation in each of these Black and White samples separately, but after preregistered exclusions, not only were the separate samples smaller than what G*Power had called for to detect a small to medium effect (*f* = .15, or *η_p_*^2^ = 0.02), but the very small number of conservatives in the Black sample (*n* = 38), indicated that we could not meaningfully examine political orientation within the Black sample as an independent factor. For this reason, we combined our Black and White participants into a total sample of 834. See Supporting Information for analyses of the separate Black and White samples. Participants were excluded if they failed (or didn’t answer) a single attention check (64 participants), answered with nothing, nonsense, or irrelevant information to an open-ended effort check (123 participants), did not identify as leaning liberal or conservative (178 participants), realized the public figure was not real (1 participant) or did not indicate at the end of the survey that they answered honestly (68 participants), leaving a final sample of 568 (290 White, 264 Black; 438 liberal, 130 conservative; *M*_age_ = 39.00, *SD*_age_ = 12.83). According to G*Power (Faul et al., 2007), this combined sample gave us the power to detect an effect size of *f* = 0.12 (η_p_^2^ = 0.01) with 80% power and α = 0.05.

#### Study 3

To allow a stronger test of effects among both liberals and conservatives, we recruited equal numbers of liberals and conservatives based on preselection data on CloudResearch, which produced an initial total of 1,231 participants. Participants were excluded if they failed (or didn’t answer) one of two attention checks (77 participants), if they failed the same effort check as in Study 2 (78 participants), if they did not identify as leaning liberal or conservative (106 participants) and if they did not indicate they were honest in their responses (80 participants), which left a final sample of 1,094 (874 White, 76 Black; 596 liberal, 498 conservative; *M*_age_ = 42.20, *SD*_age_ = 13.38). According to G*Power (Faul et al., 2007), this sample gave us the power to detect an effect size of *f* = 0.09 (η_p_^2^ = 0.01) with 80% power and α = .05.

### Procedure

Because procedures were very similar across conditions, all three are described together. If not otherwise indicated, the method and materials described were identical in each of the three studies. Any additional materials not listed can be found in copies of the surveys on OSF (https://osf.io/ag8w5/).

#### Demographics

Participants filled out basic demographic information as well as several measures of their political views. The political measure we focus on in this paper is that of “political leaning,” in which participants indicated whether they were more liberal, more conservative, or both equally on most societal topics. Participants also reported the political party in the United States they most support, as well as their position on a slider bar ranging from 0 (*liberal*) to 100 (*conservative*).

#### Manipulation

**Figure 1:**
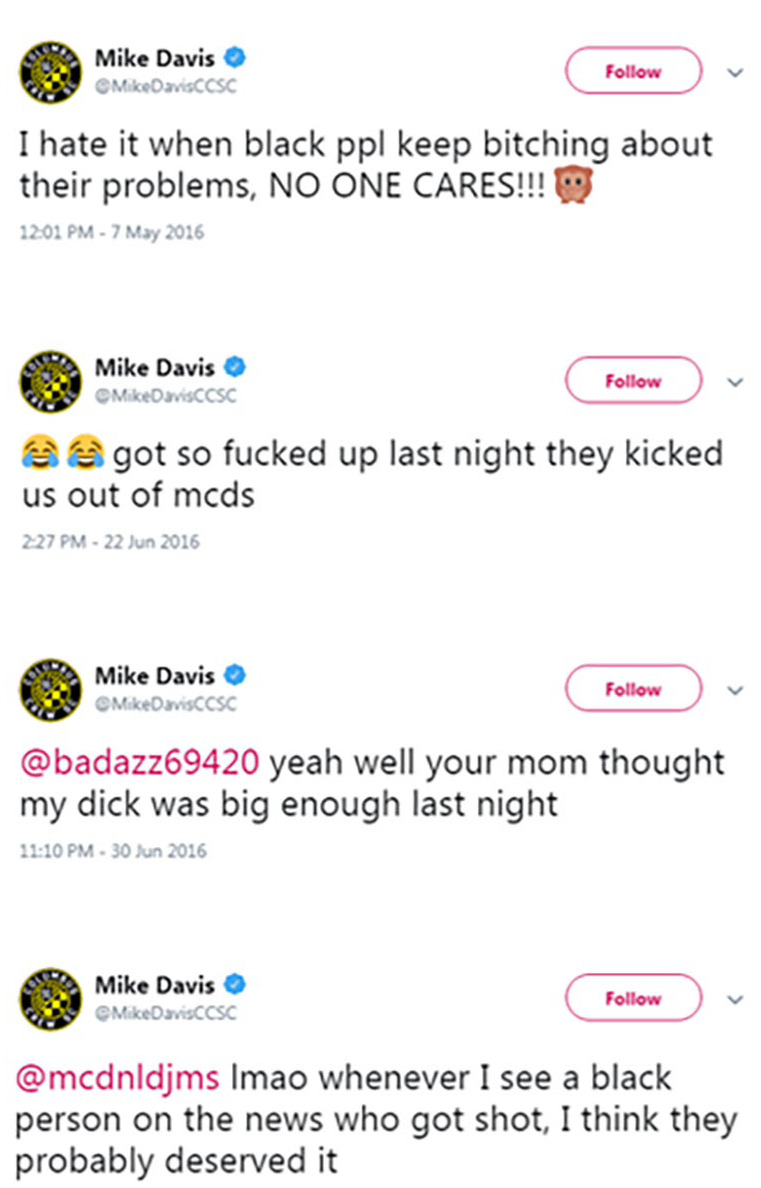
Tweets Shown to Participants (Studies 1 and 2) *Note*. In the anti-White condition, “black” was replaced with “white.”

**Figure 2:**
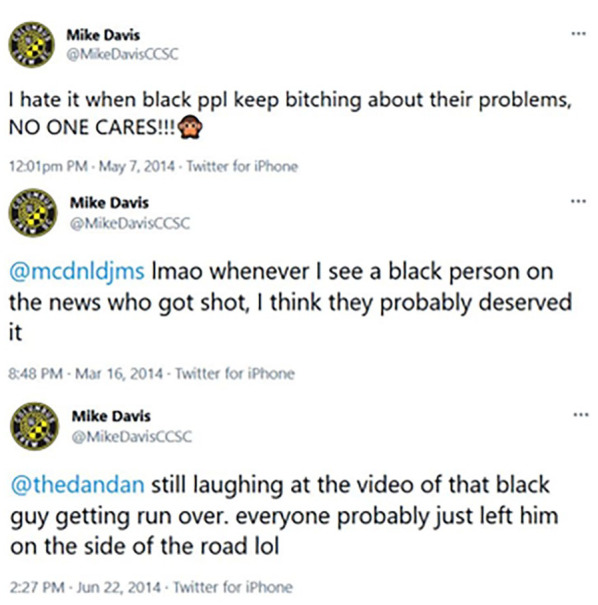
Tweets Shown to Participants (Study 3) *Note*. In the anti-White condition, “black” was replaced with “white.” In the control condition, “black” was removed.

In all studies, participants were introduced to our public figure, an ostensibly real soccer player named Mike Davis. Participants learned that Mike Davis had recently come under public scrutiny for offensive tweets uncovered from his past. The information included his age at the time of the tweets as well as how much time had passed since he posted the tweets. They were then shown screenshots of a set of tweets, some of which were derogatory toward a racial group. See Figures 1 and 2 for the appearance of the tweets in Studies 1 and 2 and Study 3, respectively. (Note that the content contains racist statements.)

All studies manipulated the amount of time that had passed (time passed; 2 years vs. 7), how old the public figure was when he posted the tweets (age at time of tweet; 16 vs. 28), and the race targeted by the tweets (targeted race; Anti-Black vs. Anti-White). Note that Wave 1 of Study 1 had an exploratory age 22 condition that was not included in Wave 2. It added little informational value and would have unnecessarily increased the required sample size (The Full 2 [Time] × 3 (Age) × 2 (Race) × 2 (Politics) factorial design would increase the study from 16 to 24 cells) and therefore will not be included in any reported analyses. Study 3 added a control condition to the targeted race variable in which no specific race was mentioned in the derogatory statements. All manipulations were randomly assigned except for targeted race in Study 1, which was split across recruitment waves.

#### Dependent Variables

##### Judgment

Our first set of items focused on how the public figure (Mike Davis) was judged in the present and the consequences participants endorsed for his past offensive tweets. All items were measured on 7-point scales. Based on exploratory factor analyses (see Supporting Information), these items were placed into two variables: consequences, which included two items on whether the figure should still be judged in the present, two items on whether the figure should be punished by his employer, and two items on whether he should apologize or resign (α = .88, 87, .90), and moral character, which included four items on the public figure’s moral character *in the present* (α = .92, .92, 93). We report results for consequences and moral character throughout the paper as our main measures of judgment. See Supporting Information for a version of the analyses with the consequences variable broken down further (present judgment, punishment, apology, resignation) and with consequences and moral character combined.

##### Subjective Circumstances

Several additional variables focused on participants’ subjective perception of the circumstances surrounding the tweets and the conclusions that arose from these views.

The *subjective time* variable used two slider items assessing how much time it felt like it had passed since the tweets were posted. The first item ranged from 0 (*Feels very recent*) to 100 (*Feels very long ago*) and the second ranged from 0 (*Feels like yesterday*) to 100 (*Feels like ancient history*). Study 2 did not include the second item. The two-item version in Studies 1 and 3 demonstrated good reliability, *r_SB_* = .95, .95 (We report reliability for all two-item measures using the Spearman-Brown coefficient, as recommended by Eisinga et al., 2013.)

*Subjective age* used two Likert-type items to ask how old if felt like the public figure was when he posted the tweets. The first item assessed agreement with the statement that “This person was quite young when they made these statements” and the second assessed agreement with “This person was old enough to know better than to make these statements” (*r_SB_* = .60, 69, .72).

The next two variables capture the degree to which people view the past offense as still pertinent and how long they believe it will remain “fresh” for present condemnation.

*Current relevance* was measured using four 7-point Likert-type items assessing agreement with a series of claims, including whether the past statements in the tweets had no bearing on the public figure in the present, whether the statements reflected beliefs deeply held by the public figure in the present, how much the past actions of the public figure reflected his current character, and the extent to which the public figure is now a different person (α = .86, .85, .92).

The *psychological statute of limitations* variable asked “How much time would have to pass before you would feel tweets like this are no longer relevant to judging that person’s character in the present?” In Study 1, this was a Likert-type scale ranging from 1 (*No time would have to pass*) to 8 (T*hese tweets would always be relevant to judging that person’s character no matter how much time has passed*), while Studies 2 and 3 had participants directly input the number of months and years that would be have to pass (from which we calculated the total number of months). A small number of participants reported extremely long-time spans (e.g., >1 trillion years) which we took to be meaningful expressions of their harsh views but which would introduce extreme outliers to the data. Rather than trimming outliers, we chose to winsorize to a realistically possible time frame such that responses >100 years were set to be equal to 100 years. Less than 1% of scores were winsorized in each study.

##### General Principles

In Study 3 only, we also included a measure of people’s agreement with general moral principles about how such online offenses should be dealt with (not specific to the target tweets they had judged previously). The *judgment principle* included two items on whether people should generally be judged in the present for past offensive public statements, two items on whether people should generally be punished by their employer for such statements, and two items on whether people should typically apologize and resign for such statements (α = .88). The *current character principle* variable asked whether participants agreed that “In general, a person’s past public offensive statements are a good indicator of their current character.” The *time principle* assessed agreement with the claim that “In general, how we judge someone for their past public offensive statements should depend on how much time has passed since they made the statements,” while the *age principle* assessed agreement about whether “In general, how we judge someone for their past public offensive statements should depend on how old they were at the time they made the statements.”

##### True Self

In Study 3, participants also assessed whether the offensive tweets represented the public figure’s “true self” using two items from Newman and colleagues (2014). A categorical item had participants choose whether the statements reflected the public figure’s true self or surface self (and a none of the above option, coded as missing data). A continuous item asked “At the time this person posted the tweets, to what extent were they being true to the deepest, most essential aspects of their being?” ranging from 1 (*Not at all*) to 9 (*Very much so*).

#### Factors That Would Change People’s Judgments

In Studies 2 and 3, we asked participants what factors would change their judgment of the public figure’s current character, with a scale ranging from −2 (*I would judge them much more harshly*) to 0 (*It would have no effect*) to 2 (*I would judge them much more charitably*). The items included factors such as if the public figure apologized before or after the tweets were discovered, if he took part in anti-discrimination causes, if he admitted his past mistakes, if he framed the tweets as harmless jokes, if he emphasized the different social norms at the time, and if he had shamed others for similar behavior.

#### Other Measures

##### Memory Checks

Participants were asked how long ago the original statements were made and how old the public figure was when he made the original statements. Study 3 added an item on which racial group was targeted by the tweets.

##### Effort Check

Studies 2 and 3 included an open-ended question that asked participants to indicate what factors they took into account when making judgments about the public figure and the tweets.

##### Honesty Check

At the end of the survey, participants were asked to indicate whether there was any reason we should not use their data in our analyses, whether that be dishonesty on their part or some other factor. All participants who indicated we should not use their data were excluded from analyses.

##### Racial Assumptions and Attitudes

We asked several additional questions about participants’ assumptions about the public figure’s race and their attitudes toward different racial groups. Most participants assumed the public figure was White, with the exception of the anti-White condition, where he was assumed to be Black. Controlling for attitudes toward Black and White people did not substantially change any of the main patterns in the results. See Supporting Information for more details.

## Results

Results were computed using SPSS 28 (IBM Corp, 2021) with confidence intervals for effect sizes obtained using CI-R2-SPSS (Wuensch, 2009).

### Memory Checks

Participants consistently demonstrated good recall of the important information we manipulated. See Table 2 for means and inferential statistics. Across all three studies, participants reported more time having passed in the 7-year condition compared with the 2-year condition, and the public figure being older in the age 28 condition compared with the age 15 condition. In Study 3, 88.5% of participants correctly recalled the race targeted (or that it was not mentioned) in the derogatory tweets.

**Table 2.** Main Effects and Interactions With Estimated Marginal Means and Standard Errors for Time Passed and Age at Time of Tweet.

Study 1							
Outcome variable	2 Years		7 Years		Main effect time passed	Main effect age at time of tweet	Time Passed × Age at Time of Tweet
	Age 16	Age 28	Age 16	Age 28			
Time memory check	1.02 (0.17)	1.38 (0.18)	4.76 (0.17)	4.77 (0.18)	*F*(1, 618) = 429.26, η_p_^2^ = .41*** [.35, .46]	*F*(1, 618) = 1.19, η_p_^2^ = .002 [<.001, .01]	*F*(1, 618) = 0.40, η_p_^2^ = .001 [<.001, .01]
Age memory check	18.47 (0.66)	27.84 (0.69)	18.36 (0.68)	26.13 (0.69)	*F*(1, 618) = 1.79, η_p_^2^ = .003 [<.001, .02]	*F*(1, 618) = 157.66, η_p_^2^ = .20*** [.15, .26]	*F*(1, 618) = 1.38, η_p_^2^ = .002 [<.001, .02]
Subjective time	42.36 (2.24)	34.95 (2.35)	54.72 (2.32)	51.40 (2.35)	*F*(1, 617) = 38.73, η_p_^2^ = .06*** [.03, .10]	*F*(1, 617) = 5.36, η_p_^2^ = .01* [<.001, .03]	*F*(1, 617) = 0.39, η_p_^2^ = .001 [<.001, .01]
Subjective age	3.93 (0.10)	5.34 (0.11)	3.59 (0.10)	5.00 (0.11)	*F*(1, 618) = 10.35, η_p_^2^ = .02** [.003, .04]	*F*(1, 618) = 182.47, η_p_^2^ = .23*** [.17, .28]	*F*(1, 618) = 0.001, η_p_^2^ < .001 [< .001, < .001]
Consequences	4.19 (0.13)	4.51 (0.13)	3.90 (0.13)	3.98 (0.13)	*F*(1, 618) = 10.01, η_p_^2^ = .02** [.002, .04]	*F*(1, 618) = 2.42, η_p_^2^ < .001 [< .001, .02]	*F*(1, 618) = 0.87, η_p_^2^ = .001 [< .001, .01]
Moral character	4.45 (0.11)	4.81 (0.11)	4.11 (0.11)	4.42 (0.11)	*F*(1, 617) = 11.27, η_p_^2^ = .02*** [.003, .04]	*F*(1, 617) = 9.55, η_p_^2^ = .02** [.002, .04]	*F*(1, 617) = 0.04, η_p_^2^ < .001 [< .001, .003]
Study 2							
Time memory check	2.25 (0.17)	2.37 (0.17)	6.36 (0.16)	6.22 (0.15)	*F*(1, 550) = 593.90, η_p_^2^ = .52*** [.47, .57]	*F*(1, 550) = 0.01, η_p_^2^ < .001 [< .001, < .001]	*F*(1, 550) = 0.62, η_p_^2^ = .001 [< .001, .01]
Age memory check	16.73 (0.31)	26.49 (0.31)	17.07 (0.28)	26.17 (0.27)	*F*(1, 545) = 0.001, η_p_^2^ < .001 [< .001, < .001]	*F*(1, 545) = 1,048.97, η_p_^2^ = .66*** [.62, .69]	*F*(1, 545) = 1.28, η_p_^2^ = .002 [< .001, .02]
Subjective time	41.18 (3.03)	46.55 (2.90)	59.81 (2.71)	47.66 (2.60)	*F*(1, 551) = 27.91, η_p_^2^ = .05*** [.02, .09]	*F*(1, 551) = 8.88, η_p_^2^ = .02*** [.002, .04]	*F*(1, 551) = 1.79 η_p_^2^ = .003 [< .001, .02]
Subjective age	3.87 (0.13)	5.56 (0.13)	3.57 (0.12)	5.19 (0.12)	*F*(1, 552) = 7.18, η_p_^2^ = .01** [.001, .04]	*F*(1, 552) = 179.44, η_p_^2^ = .25** [.19, .30]	*F*(1, 552) = 0.09, η_p_^2^ < .001 [< .001, .01]
Consequences	3.61 (0.15)	4.17 (0.14)	3.25 (0.13)	3.73 (0.13)	*F*(1, 552) = 8.51, η_p_^2^ = .02** [.002, .04]	*F*(1, 552) = 14.20, η_p_^2^ = .03*** [.01, .06]	*F*(1, 552) = 0.07, η_p_^2^ < .001 [< .001, .01]
Moral character	4.32 (0.13)	4.50 (0.12)	4.07 (0.11)	4.43 (0.11)	*F*(1, 548) = 1.77, η_p_^2^ = .003 [< .001, .02]	*F*(1, 548) = 5.21, η_p_^2^ = .01* [< .001, .03]	*F*(1, 548) = 0.59, η_p_^2^ = .001 [< .001, .01]
Study 3							
Time memory check	2.24 (0.09)	2.28 (0.09)	6.56 (0.09)	6.57 (0.09)	*F*(1, 1,069) = 2,247.68, η_p_^2^ = .68*** [.65, .70]	*F*(1, 1,069) = 0.07, η_p_^2^ < .001 [< .001, .003]	*F*(1, 1,069) = 0.05, η_p_^2^ < .001 [< .001, .002]
Age memory check	17.20 (0.30)	27.35 (0.29)	16.73 (0.29)	26.95 (0.30)	*F*(1, 1,066) = 2.18, η_p_^2^ = .002 [< .001, .01]	*F*(1, 1,066) = 1,188.64, η_p_^2^ = .53*** [.49, .56]	*F*(1, 1,066) = 0.01, η_p_^2^ < .001 [< .001, < .001]
Subjective time	39.83 (1.50)	28.60 (1.48)	55.89 (1.46)	45.34 (1.48)	*F*(1, 1,066) = 122.47, η_p_^2^ = .10*** [.07, .14]	*F*(1, 1,066) = 54.03 η_p_^2^ = .05*** [.03, .08]	*F*(1, 1,066) = 0.05, η_p_^2^ < .001 [< .001, .002]
Subjective age	3.98 (0.08)	5.85 (0.08)	3.51 (0.07)	5.38 (0.08)	*F*(1, 1,070) = 38.89, η_p_^2^ = .04*** [.02, .06]	*F*(1, 1,070) = 624.97, η_p_^2^ = .37*** [.33, .41]	*F*(1, 1,070) = 0.002 η_p_^2^ < .001 [< .001, < .001]
Consequences	3.97 (0.09)	4.45 (0.09)	3.39 (0.09)	4.01 (0.09)	*F*(1, 1,070) = 33.70, η_p_^2^ = .03*** [.01, .05]	*F*(1, 1,070) = 39.11, η_p_^2^ = .04*** [.02, .06]	*F*(1, 1,070) = 0.70, η_p_^2^ = .001 [< .001, .01]
Moral character	4.81 (0.08)	5.07 (0.08)	4.18 (0.08)	4.82 (0.08)	*F*(1, 1,069) = 33.24, η_p_^2^ = .03*** [.01, .05]	*F*(1, 1,069) = 34.84, η_p_^2^ = .03*** [.01, .05]	*F*(1, 1,069) = 6.16 η_p_^2^ = .01* [< .001, .02]

*Note*. 95% confidence intervals for effect size in square brackets.

* *p* < .05. **p < .01. ***p < .001.

### Manipulation Checks: Effects of Objective Time and Age on Subjective Time and Age

Across all three studies, our manipulations of objective time and age affected people’s subjective perceptions of time and age. See Table 2 for means and inferential statistics. Manipulation checks revealed expected patterns such that participants reported that the tweets felt further away in the 7-year condition compared with the 2-year condition, and that the public figure felt older when he had been 28 compared with 16 years old. Notably, participants also reported that tweets felt more distant when the public figure was 16, and that the public figure seemed younger when tweets occurred 7 years ago. Although these latter effects were not predicted, they suggest that perceived time and age may both inform a general sense of psychological distance.

### Hypotheses 1a and 1b: Effects of Objective Time and Age on Judgments

As we predicted, all three studies found that participants judged the public figure less harshly in the present when more time had passed and when he had been younger at the time he posted the tweets. See Table 2 for means and inferential statistics.

### Hypotheses 2a and 2b: Effects of Targeted Race and Political Leaning on Judgments

**Figure 3.**
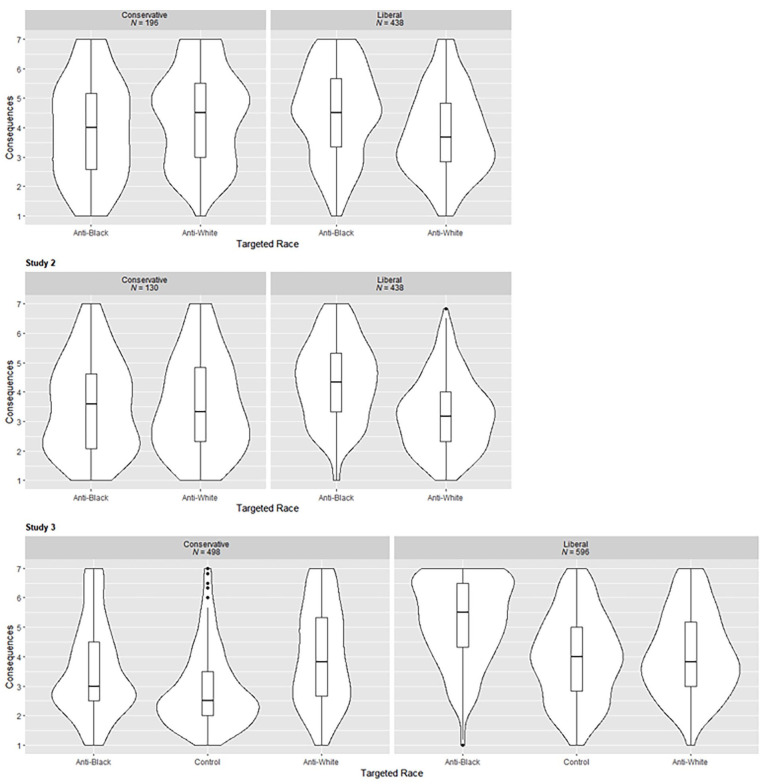
Distribution of Consequences Variable Split by Political Leaning and Targeted Race.

**Figure 4:**
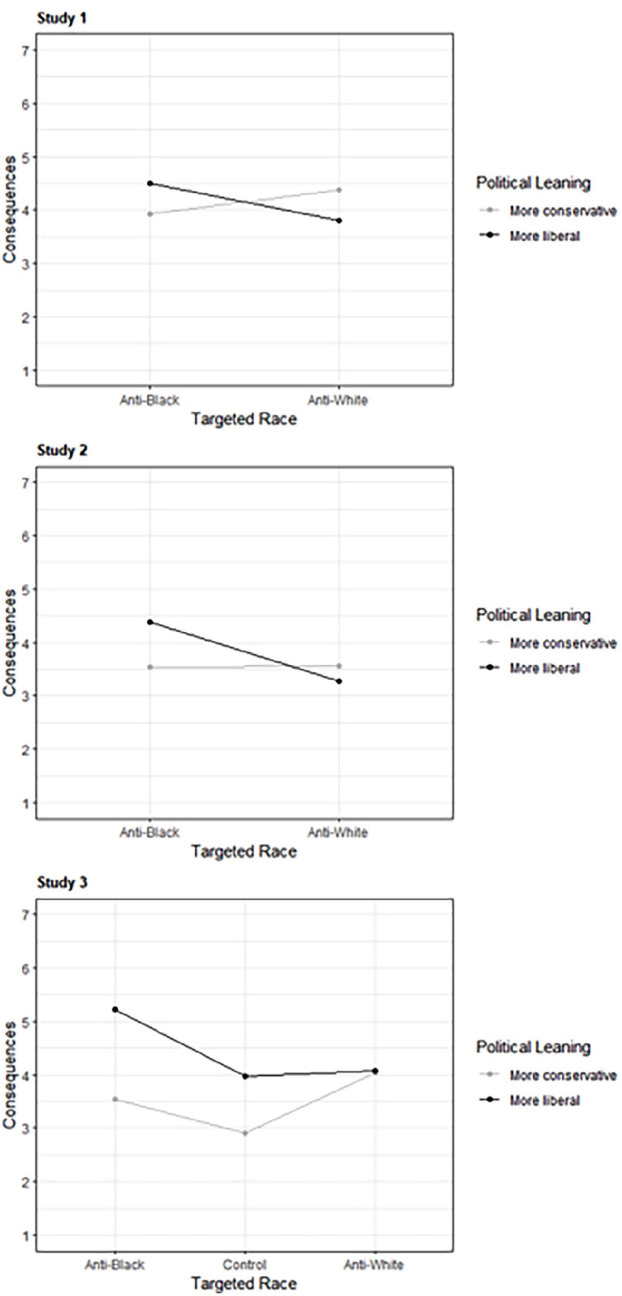
Interaction Between Political Leaning and Targeted Race for Consequences Variable

As predicted, in all three studies, liberals endorsed more severe consequences and made harsher judgments of the moral character of the public figure when he had posted anti-Black tweets compared with anti-White tweets (and compared with controls, in Study 3). Anti-White tweets and controls did not differ for either variable. In Study 3, conservatives made harsher judgments in response to anti-*White* tweets compared with anti-*Black* tweets (and controls) for both the consequences and moral character variables. Anti-Black tweets were still judged more harshly than controls, however. In Study 1, conservatives endorsed more severe consequences for anti-White tweets but did not differ for moral character, and in Study 2 conservatives did not show differences for either variable. This may reflect lower power in the first two studies where we did not recruit equal numbers of conservatives. See Table 3 for means and inferential statistics and Figure 3 for the distribution of the consequences variable. The interaction is illustrated in Figure 4.

**Table 3.** Main Effects and Interactions With Estimated Marginal Means and Standard Errors for Political Leaning and Targeted Race.

Study 1									
Outcome variable	Conservative			Liberal			Main effect political leaning	Main effect targeted race	Political Leaning × Targeted Race
	Anti-Black	Anti-White		Anti-Black	Anti-White				
Consequence	3.93_aa_ (0.15)	4.37_ba_ (0.15)		4.50_ab_ (0.11)	3.79_bb_ (0.10)		*F*(1, 618) = 0.004, η_p_^2^ < .001 [< .001, < .001]	*F*(1, 618) = 1.05, η_p_^2^ = .002 [< .001, .01]	*F*(1, 618) = 19.48, η_p_^2^ = .03*** [.01, .06]
Moral character	4.27_aa_ (0.13)	4.44_aa_ (0.13)		4.27_ab_ (0.08)	4.82_ba_ (0.09)		*F*(1, 617) = 3.01, η_p_^2^ = .01† [< .001, .02]	*F*(1, 617) = 3.15, η_p_^2^ = .01† [< .001, .02]	*F*(1, 617) = 11.07, η_p_^2^ = .02*** [.003, .04]
Current relevance	4.20_aa_ (0.13)	4.82_ba_ (0.14)		4.70_ab_ (0.09)	4.26_bb_ (0.09)		*F*(1, 618) = 0.08, η_p_^2^ < .001 [< .001, .06]	*F*(1, 618) = 0.66, η_p_^2^ = .001 [< .001, .01]	*F*(1, 618) = 21.62, η_p_^2^ = .03*** [.01, .07]
Statute of limitations	4.55_aa_ (0.19)	5.54_ba_ (0.20)		5.59_ab_ (0.14)	5.42_ab_ (0.12)		*F*(1, 618) = 7.82, η_p_^2^ = .01** [.001, .04]	*F*(1, 618) = 6.02, η_p_^2^ = .01* [< .001, .03]	*F*(1, 618) = 12.32, η_p_^2^ = .02*** [.003, .05]
Subjective time	54.89_aa_ (2.69)	42.82_ba_ (2.75)		40.22_ab_ (1.92)	45.50_bb_ (1.72)		*F*(1, 617) = 6.71, η_p_^2^ = .01* [.001, .03]	*F*(1, 617) = 2.15, η_p_^2^ = .003 [< .001, .02]	*F*(1, 617) = 14.04, η_p_^2^ = .02*** [.01, .05]
Subjective age	4.14_aa_ (0.12)	4.71_ba_ (0.12)		4.65_ab_ (0.09)	4.36_bb_ (0.08)		*F*(1, 618) = 0.55, η_p_^2^ = .001 [< .001, .004]	*F*(1, 618) = 1.81, η_p_^2^ = .003 [< .001, .02]	*F*(1, 618) = 16.70, η_p_^2^ = .03*** [.01, .06]
Study 2									
Consequence	3.53_aa_ (0.18)	3.58_aa_ (0.17)		4.37_ab_ (0.09)	3.28_ba_ (0.09)		*F*(1, 552) = 3.97, η_p_^2^ = .01* [< .001, .03]	*F*(1, 552) = 14.65, η_p_^2^ = .03*** [.01, .06]	*F*(1, 552) = 17.20, η_p_^2^ = .03*** [.01, .06]
Moral character	4.20_aa_ (0.15)	4.26_aa_ (0.14)		4.82_ab_ (0.08)	4.04_ba_ (0.08)		*F*(1, 548) = 2.88, η_p_^2^ = .01† [< .001, .02]	*F*(1, 548) = 9.25, η_p_^2^ = .02** [.002, .04]	*F*(1, 548) = 12.91, η_p_^2^ = .02*** [.004, .05]
Current relevance	4.18_aa_ (0.16)	4.10_aa_ (0.15)		4.83_ab_ (0.15)	3.98_ba_ (0.08)		*F*(1, 552) = 4.75, η_p_^2^ = .01* [< .001, .03]	*F*(1, 552) = 14.69, η_p_^2^ = .03* [.01, .06]	*F*(1, 552) = 9.73, η_p_^2^ = .02** [.002, .04]
Statute of limitations	87.49_aa_ (20.98)	73.09_aa_ (20.11)		125.92_aa_ (11.34)	85.39_ba_ (10.81)		*F*(1, 525) = 2.36, η_p_^2^ = .004 [< .001, .02]	*F*(1, 525) = 2.77, η_p_^2^ = .01† [< .001, .02]	*F*(1, 525) = 0.63, η_p_^2^ = .001 [< .001, .02]
Subjective time	49.74_aa_ (3.63)	44.98_aA_ (3.39)		38.72_ab_ (1.93)	51.77_bA_ (1.83)		*F*(1, 551) = 0.56, η_p_^2^ = .001 [< .001, .01]	*F*(1, 551) = 2.16, η_p_^2^ = .004 [< .001, .02]	*F*(1, 551) = 10.00, η_p_^2^ = .02** [.003, .05]
Subjective age	4.50_aa_ (0.16)	4.41_aa_ (0.15)		4.94_ab_ (0.09)	4.34_ba_ (0.08)		*F*(1, 552) = 2.31, η_p_^2^ = .004 [< .001, .02]	*F*(1, 552) = 7.87, η_p_^2^ = .01** [.001, .04]	*F*(1, 552) = 4.26, η_p_^2^ = .01** [< .001, .03]
Study 3									
	Conservative			Liberal			Main effect political leaning	Main effect targeted race	Political Leaning × Targeted Race
	Anti-Black	Control	Anti-White	Anti-Black	Control	Anti-White			
Consequence	3.56_aa_ (0.11)	2.93_ba_ (0.11)	4.00_ca_ (0.12)	5.22_ab_ (0.11)	3.94_bb_ (0.10)	4.09_ba_ (0.10)	*F*(1, 1070) = 110.60, η_p_^2^ = .09*** [.06, .13]	*F*(2, 1070) = 40.96, η_p_^2^ = .07*** [.04, .10]	*F*(2, 1070) = 26.61, η_p_^2^ = .05*** [.02, .07]
Moral character	4.44_aa_ (0.10)	4.13_ba_ (0.10)	4.83_ca_ (0.10)	5.49_ab_ (0.10)	4.77_bb_ (0.08)	4.66_ba_ (0.09)	*F*(1, 1,069) = 44.40, η_p_^2^ = .04*** [.02, .07]	*F*(2, 1,069) = 15.19, η_p_^2^ = .03*** [.01, .05]	*F*(2, 1,069) = 21.55, η_p_^2^ = .04*** [.02, .06]
Current relevance	4.13_aa_ (0.11)	3.76_ba_ (0.11)	4.75_ca_ (0.11)	5.31_ab_ (0.11)	4.46_bb_ (0.09)	4.46_bb_ (0.10)	*F*(1, 1,070) = 39.46, η_p_^2^ = .04*** [.02, .06]	*F*(2, 1,070) = 20.48, η_p_^2^ = .04*** [.02, .06]	*F*(2, 1,070) = 26.27, η_p_^2^ = .05*** [.02, .07]
Statute of limitations	98.37_aa_ (14.82)	89.03_aa_ (15.16)	144.31_ba_ (15.57)	201.89_ab_ (15.14)	120.82_ba_ (13.10)	120.47_ba_ (13.42)	*F*(1, 1,039) = 9.77, η_p_^2^ = .01** [.001, .02]	*F*(2, 1,039) = 4.93, η_p_^2^ = .01** [.001, .02]	*F*(2, 1,039) = 9.34, η_p_^2^ = .02*** [< .001, .04]
Subjective time	47.33_aa_ (1.86)	49.28_aa_ (1.88)	39.33_ba_ (1.94)	29.85_ab_ (1.87)	44.07_bb_ (1.64)	44.63_bb_ (1.68)	*F*(1, 1,066) = 15.32, η_p_^2^ = .01*** [.004, .03]	*F*(2, 1,066) = 10.09, η_p_^2^ = .02*** [.01, .04]	*F*(2, 1,066) = 19.16, η_p_^2^ = .04*** [.02, .06]
Subjective age	4.34_aa_ (0.09)	4.31_aa_ (0.10)	4.89_ba_ (0.10)	5.29_ab_ (0.10)	4.64_bb_ (0.08)	4.60_bb_ (0.09)	*F*(1, 1,070) = 19.43, η_p_^2^ = .02*** [.06, .04]	*F*(2, 1,070) = 8.10, η_p_^2^ = .02*** [.003, .03]	*F*(2, 1,070) = 22.02, η_p_^2^ = .04*** [.02, .06]
Judgment principle	3.04_aa_ (0.09)	2.93_aa_ (0.09)	3.48_ba_ (0.09)	4.60_ab_ (0.09)	4.02_bb_ (0.08)	3.88_bb_ (0.08)	*F*(1, 1,070) = 205.74, η_p_^2^ = .16*** [.12, .20]	*F*(2, 1,070) = 7.87, η_p_^2^ = .01***, [.003, .03]	*F*(2, 1,070) = 22.09, η_p_^2^ = .04*** [.02, .06]
Current character principle	3.61_aa_ (0.12)	3.28_ba_ (0.12)	4.13_ca_ (0.13)	4.64_ab_ (0.12)	4.20_bb_ (0.11)	4.11_ba_ (0.11)	*F*(1, 1,069) = 46.10, η_p_^2^ = .04*** [.02, .07]	*F*(2, 1,069) = 7.45, η_p_^2^ = .01*** [.003, .03]	*F*(2, 1,069) = 12.26, η_p_^2^ = .02*** [.01, .04]
Time principle	4.70_aa_ (0.11)	4.86_aa_ (0.12)	4.67_aa_ (0.12)	4.32_ab_ (0.12)	4.69_ba_ (0.10)	4.68_ba_ (0.10)	*F*(1, 1,069) = 3.75, η_p_^2^ = .004† [< .001, .01]	*F*(2, 1,069) = 2.91, η_p_^2^ = .01† [< .001, .02]	*F*(2, 1,069) = 1.56, η_p_^2^ = .003 [< .001, .01]
Age principle	4.97_aa_ (0.12)	5.04_aa_ (0.12)	4.94_aa_ (0.12)	4.54_ab_ (0.12)	5.09_ba_ (0.10)	4.94_ba_ (0.11)	*F*(1, 1,070) = 1.96, η_p_^2^ = .002 [< .001, .01]	*F*(2, 1,070) = 3.59, η_p_^2^ = .01* [< .001, .02]	*F*(2, 1,070) = 2.59, η_p_^2^ = .01† [< .001, .02]
True self-continuous	5.53_aa_ (0.16)	4.96_ba_ (0.16)	5.95_aA_ (0.17)	6.32_ab_ (0.16)	5.58_bb_ (0.14)	5.52_bA_ (0.15)	*F*(1, 1,069) = 6.18, η_p_^2^ = .01* [< .001, .02]	*F*(2, 1,069) = 9.15, η_p_^2^ = .02*** [.004, .03]	*F*(2, 1,069) = 8.48, η_p_^2^ = .02*** [.004, .03]

*Note*. 95% confidence intervals for effect size in square brackets. Different subscript letters across means denote a significant difference. First subscript refers to simple effects of targeted race within both conservatives and liberals. Second subscript refers to simple effects of political leaning for anti-Black, anti-White, and (for Study 3) control tweets. Marginal differences are denoted by the same letter in all caps.

* *p* < .05. **p < .01. ***p < .001. †*p* < .10.

### Hypotheses 3a and 3b: Effects of Targeted Race and Political Leaning on Subjective Circumstances

Across all three studies, in line with our predictions, liberals viewed the subjective circumstances in the way that would reflect most poorly on the public figure when he had posted anti-Black tweets compared with anti-White tweets (and controls in Study 3). See Table 3 for means and inferential statistics. Anti-Black tweets felt subjectively closer in time, and the public figure seemed older when he posted them, independent of objective time. Furthermore, liberals viewed the past offense as more relevant to the present and reported a lengthier psychological statute of limitations before they would stop judging the target for the offense. Conservatives, by contrast, showed the parallel pattern in Studies 1 and 3, such that they judged the anti-*White* tweet as subjectively closer, the target as subjectively older, and the tweets as more currently relevant and with a longer statute of limitations compared with anti-*Black* tweets (and controls in Study 3), though again this was not found in Study 2. (Study 2 found an interaction, but the simple main effect was only significant for liberals.)

### Hypotheses 4a and 4b: Path Analysis

**Figure 5:**
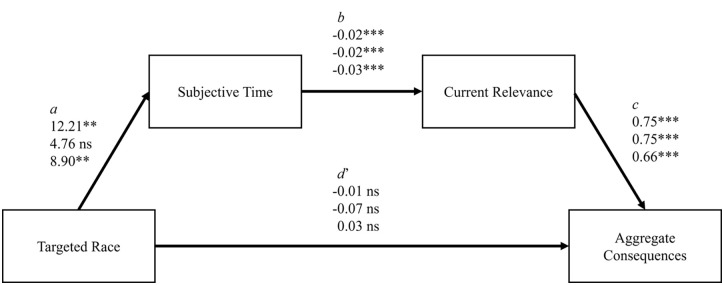
Regression Coefficients for Path Analysis (Conservatives). *Note*. Presents each coefficient for Studies 1, 2, and 3, respectively. Targeted race was coded 0 = Anti-White, 1 = Anti-Black. *d’* represents the direct effect of targeted race on the aggregate consequence variable.

**Figure 6:**
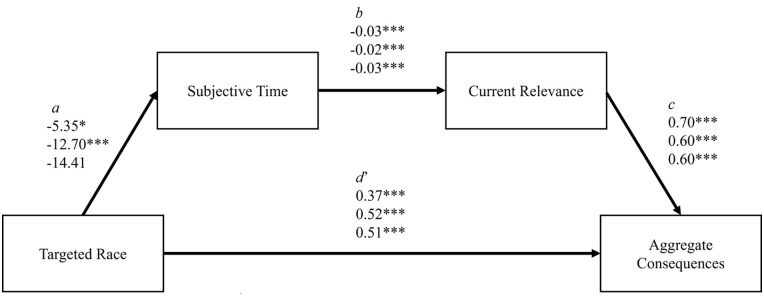
Regression Coefficients for Path Analysis (Liberals). *Note*. Presents each coefficient for Studies 1, 2, and 3, respectively. Targeted race was coded 0 = Anti-White, 1 = Anti-Black. *d’* represents the direct effect of targeted race on the aggregate consequence variable.

We predicted (preregistered in Study 3) that the interaction between political leaning and targeted race would predict subjective time, which would in turn predict current relevance, which would in turn predict consequences. Across all three studies, we tested this relationship by running a moderated mediation using a custom model in PROCESS (Hayes, 2017), conducted with 5,000 bootstrap samples. Although cross-sectional mediation analysis can produce biased estimates (Maxwell & Cole, 2007; Maxwell et al., 2011) and establishing a causal chain of several mediators may require a more complex strategy of several studies that manipulate each variable (Spencer et al., 2005), we offer a correlational analysis that may inform future experimental tests of this process. Targeted race was entered as the predictor variable, political leaning as the moderator, subjective time as the first mediator, current relevance as the second mediator, and the consequence measure as the outcome variable. All three studies found that each step was significant as well as the overall index of moderated mediation. The separate indirect effects for liberals and conservatives were not significant across all studies, but they were both found in Study 3 when we had a large sample of both political groups. To illustrate the model, a liberal who sees anti-Black tweets will see the tweets as closer in time, and therefore more relevant, and therefore give harsher judgments to the public figure, while a conservative who sees anti-Black tweets will see the tweets as *further* in time, and therefore *less* relevant, and therefore give *more forgiving* judgments of the public figure. The model is illustrated in Figures 5 and 6 for conservatives and liberals respectively. Values and inferential statistics can be found in Table 4.

**Table 4:** Inferential Statistics for Path Models.

Study 1					
Subjective time model			Subjective age model		
	Point estimate	95% CI		Point estimate	95% CI
Political Leaning × Targeted Race → Subjective Time (*a*)	−17.56	−27.06, −8.05	Political Leaning × Targeted Race → Subjective Age (*a*)	0.74	0.25, 1.23
Subjective Time → Current Relevance (*b*)	−0.03	−0.03, −0.02	Subjective Age → Current Relevance (*b*)	0.54	0.48, 0.60
Current Relevance → Consequences (*c*)	0.72	0.65, 0.80	Current Relevance → Consequences (*c*)	0.72	0.64, 0.79
Direct Effect (*d’*)	0.35	−0.04, 0.74	Direct Effect (*d’*)	0.37	−0.02, 0.75
Index of Moderated Mediation	0.34	0.14, 0.55	Index of Moderated Mediation	0.29	0.10, 0.48
Conservative Indirect Effect	−0.24	−0.41, −0.08	Conservative Indirect Effect	−0.18	−0.33, −0.05
Liberal Indirect Effect	0.10	−0.0002, 0.21	Liberal Indirect Effect	0.10	−0.01, 0.22
Study 2					
Political Leaning × Targeted Race → Subjective Time (*a*)	−17.46	−29.15, −5.77	Political Leaning × Targeted Race → Subjective Age (*a*)	0.49	−0.10, 1.08
Subjective Time → Current Relevance (*b*)	−0.02	−0.03, −0.02	Subjective Age → Current Relevance (*b*)	0.45	0.39, 0.51
Current Relevance → Consequences (*c*)	0.63	0.55, 0.72	Current Relevance → Consequences (*c*)	0.63	0.55, 0.71
Direct Effect (*d’*)	0.53	0.12, 0.95	Direct Effect (*d’*)	0.58	0.17, 0.98
Index of Moderated Mediation	0.25	0.08, 0.44	Index of Moderated Mediation	0.14	−0.02, 0.30
Conservative Indirect Effect	−0.07	−0.22, 0.09	Conservative Indirect Effect	0.05	−0.09, 0.19
Liberal Indirect Effect	0.18	0.10, 0.44	Liberal Indirect Effect	0.18	0.10, 0.30
Study 3					
Political Leaning × Targeted Race → Subjective Time (*a*)	−23.32	−30.94, −15.69	Political Leaning × Targeted Race → Subjective Age (*a*)	1.19	0.73, 1.64
Subjective Time → Current Relevance (*b*)	−0.03	−0.04, −0.03	Subjective Age → Current Relevance (*b*)	0.58	0.52, 0.63
Current Relevance → Consequences (*c*)	0.63	0.56, 0.70	Current Relevance → Consequences (*c*)	0.61	0.54, 0.68
Direct Effect (*d’*)	0.47	0.14, 0.80	Direct Effect (*d’*)	0.48	0.15, 0.81
Index of Moderated Mediation	0.47	0.30, 0.67	Index of Moderated Mediation	0.42	0.25, 0.62
Conservative Indirect Effect	−0.18	−0.31, −0.06	Conservative Indirect Effect	−0.18	−0.31, −0.06
Liberal Indirect Effect	0.29	0.18, 0.42	Liberal Indirect Effect	0.24	0.12, 0.37

We also tested an identical path model that included subjective age in the place of subjective time. This model was supported in Studies 1 and 3, with all paths significant as well as the index of moderated mediation. The index was not significant in Study 2. Based on this model, a liberal who sees anti-Black tweets will see the public figure as older at the time of the tweets, and in turn judge his tweets as more currently relevant, and therefore judge him more harshly, whereas a conservative who sees the same tweets will see the public figure as *younger*, and therefore his tweets as *less* relevant, and therefore judge him *less harshly*.

### Hypotheses 5a and 5b: Effects of Targeted Race and Political Leaning on General Principles

As predicted, liberals consistently endorsed more judgemental and punitive general principles after having encountered anti-Black tweets compared with anti-White tweets and controls. When exposed to anti-Black tweets, liberals endorsed general principles that indicated people who posted offensive statements should be judged more, that the tweets are more reflective of their current character, that time is less important, and that age is less important as well. Conservatives, by contrast, endorsed harsher principles when exposed to anti-*White* tweets compared with anti-Black tweets and controls, but only with regard to judgment and character. Overall, these findings suggest that people not only make different judgments of the particular situation but may shift their general beliefs to suit their current judgments. See Table 3 for means and inferential statistics.

### True Self

For both the continuous (Table 3) and categorical (Supporting Information, Supplemental Table S9) measures, liberals were more inclined to say that the offensive tweets reflected the public figure’s true self when the statements expressed anti-Black sentiment compared with anti-White and control. Similar to the subjective circumstances variables, this appears to be a bias toward whatever reflects more poorly on the public figure in the case of anti-Black tweets. Conservatives did not show any differences for the categorical measure, and for the continuous measure only gave higher true self-ratings for both anti-Black and anti-White compared with control.

### Participant Race in Study 2

Though we were unable to follow through on our initial plan for Study 2 to examine separate samples of Black and White participants, we sought to examine the contribution of race in another way. In the combined sample in Study 2, excluding participants who did not identify as Black or White (*n* = 14), we ran a Participant Race × Targeted Race ANOVA controlling for political leaning.^1^ See Table 5 for means and inferential statistics. Participants overall responded more harshly to anti-Black tweets compared with anti-White tweets, and Black participants gave harsher responses overall than White participants. We also found a significant interaction for consequences, moral character, current relevance, and subjective time. For all variables, only Black participants differed across tweets, seeing anti-Black statements as closer and more relevant.

**Table 5:** Main Effects and Interactions With Estimated Marginal Means and Standard Errors for Participant Race and Targeted Race While Controlling for Political Leaning (Study 2)

Outcome variable	Black participants		White participants		Main effect participant race	Main effect targeted race	Participant Race × Targeted Race
	Anti-Black	Anti-White	Anti-Black	Anti-White			
Consequences	4.64_aa_ (0.13)	3.24_ba_ (0.12)	3.62_ab_ (0.12)	3.36_aa_ (0.11)	*F*(1, 549) = 14.79, η_p_^2^ = .03*** [.01, .06]	*F*(1, 549) = 51.00, η_p_^2^ = .09*** [.05, .13]	*F*(1, 549) = 23.75, η_p_^2^ = .04*** [.01, .08]
Moral character	5.00_aa_ (0.11)	3.96_ba_ (0.11)	4.27_ab_ (0.10)	4.13_aa_ (0.10)	*F*(1, 545) = 7.70, η_p_^2^ = .01** [.001, .04]	*F*(1, 545) = 35.02, η_p_^2^ = .06*** [.03, .10]	*F*(1, 545) = 20.58, η_p_^2^ = .04*** [.01, .07]
Current relevance	5.06_aa_ (0.11)	3.91_ba_ (0.11)	4.27_ab_ (0.11)	4.07_aa_ (0.10)	*F*(1, 549) = 8.96, η_p_^2^ = .02** [.002, .04]	*F*(1, 549) = 40.79, η_p_^2^ = .07*** [.03, .11]	*F*(1, 549) = 20.18, η_p_^2^ = .04*** [.01, .07]
Statute of limitations	135.12_aa_ (14.97)	78.78_ba_ (14.17)	105.92_aa_ (14.22)	83.05_aa_ (13.61)	*F*(1, 523) = 0.76, η_p_^2^ = .001 [< .001, .02]	*F*(1, 523) = 7.74, η_p_^2^ = .02** [.001, .04]	*F*(1, 523) = 1.38, η_p_^2^ = .003 [< .001, .02]
Subjective time	34.04_aa_ (2.62)	47.49_bA_ (2.49)	49.77_ab_ (2.53)	53.75_aA_ (2.37)	*F*(1, 548) = 19.10, η_p_^2^ = .03*** [.01, .07]	*F*(1, 548) = 12.15, η_p_^2^ = .02*** [.004, .05]	*F*(1, 548) = 3.59, η_p_^2^ = .01† [< .001, .03]
Subjective age	5.14_aa_ (0.13)	4.58_ba_ (0.13)	4.59_ab_ (0.13)	4.08_bb_ (0.12)	*F*(1, 549) = 17.03, η_p_^2^ = .03*** [.01, .06]	*F*(1, 549) = 17.96, η_p_^2^ = .03*** [.01, .07]	*F*(1, 549) = 0.03, η_p_^2^ < .001 [<.001, .002]

*Note*. 95% confidence intervals for effect size in square brackets. Different subscript letters across means denotes a significant difference. First subscript refers to simple effects of targeted race for Black participants and White participants. Second subscript refers to simple effects of participant race for anti-Black and anti-White tweets. Marginal differences are denoted by the same letter in all caps.

* *p* < .05. **p < .01. ***p < .001. †*p* < .10.

### Factors That Would Change People’s Judgments

In Studies 2 and 3, we included exploratory questions about what factors people would consider that would make a past online transgression more or less forgivable. In Table 6, we report descriptive means and standard deviations of the factors that participants reported would contribute to greater condemnation or redemption for the target. Participants reported that the presence of an apology, whether the public figure had contributed time and money to anti-discrimination causes, and whether the public figure had acknowledged their past mistakes and personal growth since the incident could reduce the harshness of judgments. Notably, the factor most likely to make the situation worse was whether the public figure had shamed others for similar tweets, something that likely invoked a sense of hypocrisy.

**Table 6.** Means and Standard Deviations for Factors That Would Change People’s Judgments.

Item	Study 2	Study 3
“If I learned this person publicly apologized after the tweets were discovered.”	0.81 (0.84)	0.75 (0.83)
“If I learned this person had already publicly apologized before the tweets were discovered.”	1.26 (0.88)	1.38 (0.80)
“If I learned this person had actively contributed time and money to anti-discrimination causes in the recent past.”	1.10 (0.96)	1.15 (0.93)
“If I learned this person publicly acknowledged their past mistakes and personal growth.”	1.28 (0.82)	1.35 (0.76)
“If I learned this person emphasized how harmless jokes on twitter often get misunderstood.”	−0.53 (1.20)	−0.64 (1.16)
“I learned this person had deleted these tweets long before they came to light.”	0.19 (1.00)	0.20 (0.98)
“If I learned that this person emphasized how social norms were different back when he sent those tweets.”	−0.53 (1.12)	−0.55 (1.05)
“If I learned this person had publicly shamed others for similar kinds of tweets.”	−0.81 (1.19)	−1.03 (1.10)

*Note*. Scale ranges from −2.00 to 2.00. Negative values indicate that the participant would judge the public figure more harshly, while positive values indicate that the participant would judge them more charitably.

## Discussion

Across three studies with a total of 2,296 participants, we have illustrated how people consider multiple factors and engage in a number of different cognitive processes when judging others for past offensive statements in the public sphere. Participants did consistently consider some basic factors that would be expected in principle to inform how harshly to judge a past offense in the present: They weighed the passage of time, judging the target less harshly when the tweets were further away. As well, they were concerned with how old the public figure was when he posted the offensive statements online, showing more forgiveness toward younger offenders.

At the same time, partisan sensibilities played a central role when it came to forgiving or condemning the public figure. Independent of the timing or age of the offender, liberals viewed the public figure who made anti-Black past statements to be more blameworthy in the present, while conservatives showed more present condemnation of the target who made anti-White statements in the past. The control group in Study 3 added some clarity regarding the direction of effects: liberals saw anti-Black tweets as especially blameworthy and did not distinguish anti-White tweets from the more generic (no race mentioned) offensive tweets on any dependent measure. In contrast, conservatives did judge both anti-White and anti-Black tweets more harshly than the controls, but only shifted their other judgments of subjective circumstances in a way that kept anti-White tweets as more currently relevant than anti-Black or control offenses.

Of course, part of this pattern simply reflects the fact that liberals and conservatives found the two offenses differently blameworthy regardless of the passage of time or age of the offender. This is consistent with past work by Dias and colleagues (2022) who found that both sides of the political spectrum engage in cancelation but for different offenses. However, partisans also shifted their subjective perceptions of these very circumstances—how distant in time the offense felt and how old the offender seemed—in ways that supported the view that these distant transgressions were either still highly relevant in the present and worthy of sanction today, or that they were foibles of the young and foolish offender in the remote past, and irrelevant to the offender’s current character. Specifically, liberals reported the anti-Black tweets to feel closer in subjective time and the offender as seeming older at the time whereas conservatives tended to relegate those tweets to the remote subjective past and viewed the offender as subjectively young, with the reverse pattern appearing for anti-White tweets. Moderated mediation analyses supported the proposed theoretical causal chain whereby motivated shifts in subjective perceptions of circumstances (timing and age) justify judgments of greater or lesser relevance in the present, in turn supporting more or less current condemnation. Of course, path analyses in the current paper must be interpreted cautiously as cross-sectional mediation analysis can produce estimates that are positively or negatively biased under conditions of both partial and full mediation (Maxwell & Cole, 2007; Maxwell et al., 2011). As well, these correlational analyses cannot establish strong support for any causal mechanism. Establishing a full causal chain by manipulating each subsequent variable in the model (Spencer et al., 2005), is beyond the scope of the current manuscript, but results are consistent with past experimental research in other relational contexts that demonstrated causal links between subjective time, perceived relevance, and harsh present judgment (Cortes et al., 2018; Dawson et al., 2022). To our knowledge, less research has been done to investigate these causal links for subjective age; current analyses supports related past research on perceived age (Rattan et al., 2012) and may be generative of future investigation of how subjective age of offenders conditions judgment.

We reasoned that time and age considerations are two principles relevant to judging distant past offenses and demonstrated across studies that people consider these principles but also shift their meaning in systematic ways. This extends past research on political casuistry and motivated use of moral principles (Knowles & Ditto, 2012; Uhlmann et al., 2009) by demonstrating that not only do people sometimes shift the principle they rely on to arrive at their preferred moral judgment, they may also change their perception of the very circumstances (time, age) relevant to that principle. In Study 3, we examined principles further by asking participants not only about their judgments of the target offender but also their endorsement of different principles of judgment. Participants shifted the general principles they supported largely in tandem with their judgments of the salient offense. In line with past research, these findings imply that people’s adoption of moral principles is quite flexible. It is possible that these shifts in principle are only a fleeting consequence of the study context since they were asked shortly after the questions about the specific offense. However, it also raises the possibility that perceivers’ moral judgments in later situations could be influenced by the principles they adopted in the current case.

### Limitations

#### Self-Report

Respondents self-reported their judgments about the online offenses, which may not fully reflect their corresponding behavior were they to encounter such an offense in real time in an online context. We cannot say whether people’s perceptions of the public figure in this study would translate to condemnation in an online context, both because of the well-known gap between self-reported attitudes and actual behavior (Webb & Sheeran, 2006) and because the online context would involve additional factors (e.g., virality and controversy of the tweet and its condemnation, reputational or self-presentational goals, anonymity, etc.) that could encourage or inhibit online outrage. Conducting experimental research of this nature in more realistic online contexts can be methodologically and ethically complex and could benefit from simulation tools such as the Mock Social Media Tool (Jagayat et al., 2021); this approach could be complemented by research examining these processes “in the wild” using real-world Twitter data.

Nonetheless, it is arguably also important to examine these psychological processes offline to gather richer information about people’s (self-reported) moral reasoning under controlled conditions as we have here. The current studies, therefore, point to numerous future research directions to better understand the way these phenomena play out publicly and how they may compare to people’s private opinions.

#### Generalizability

Although the current studies recruited relatively large and diverse samples of American participants including additional recruitment of Black (Study 2) and liberal/conservative (Study 3) respondents, they are nonetheless limited to the U.S. context and all focus on a similar set of racist offenses made by one specific public figure (male athlete). The fact that participants made different assumptions about the public figure’s race depending on the statement content could have influenced responses. We would expect that the presumed racial identity of the public figure and content of the tweet would produce similar judgments, and these two possible influences cannot be disentangled here. Future research could examine these factors separately as well as a wider range of transgression types and offender characteristics (e.g., the race, gender, and status of the offending target), and expand to study different countries with distinct political landscapes.

### Future Directions

Although the current research explores several factors that affect condemnation or forgiveness for a past online offense, there are additional factors that could be examined in future research. Indeed, some of these factors were explored descriptively by asking participants what actions would tend to reduce or heighten the harshness of their current judgment of the offender. Respondents indicated that apology, acknowledgment of wrongdoing, and evidence of personal growth would reduce their current condemnation.

Respondents’ intuition about the benefits of apology is consistent with past research documenting its reparative effects (Kammrath & Peetz, 2012; Schumann & Dragotta, 2020). Of course, not all apologies are viewed as equally helpful, and participants noted that an apology that came after the public outcry would be less convincing than one that came unbidden. Although this distinction is understandable since publicly demanded apologies may sometimes be inauthentic, in real-world social media contexts it may disqualify a great many public apologies that occur after long-forgotten offenses come to light. In a similar vein, evidence of personal growth can mitigate the harshness of judgment for past transgressions. When someone commits a transgression, they are often motivated to act in a moral manner to compensate (Jordan et al., 2011; West & Zhong, 2015). We know little, however, about whether and when these reparative acts function to absolve the original harm in the eyes of others, and when the growth may be dismissed as inauthentic or performative.

Although actions like apology, acknowledgment, and growth may increase people’s willingness to grant redemption for past moral offenses, other factors may heighten condemnation. For example, respondents indicated that minimizing the offense (as just a joke or acceptable by past standards), and hypocrisy (e.g., shaming someone else for an offense they too have committed) exacerbates the harshness of moral judgments. These factors should be examined systematically in future research to determine whether their actual effects correspond with respondents’ hypothetical assessments.

## Conclusion

The current research provides one piece of the puzzle when it comes to judgments of past offenses from public figures that have resurfaced in the present, something that is likely to become increasingly common as people live more and more of their lives online. People take a number of factors into account when determining the level of blame and appropriate punishment for past offensive statements: How long ago it happened and the age of the offender matter, but so does the nature of the offense (anti-Black vs. anti-White tweets in this case) and the degree to which it offends certain political sensibilities. However, although the current research answers several questions, it leaves open many more if we are to develop a thorough scientific understanding of the psychology of what may be called cancel culture or accountability culture, or more fundamentally, if we are to understand how people reason about the nature of morality, growth, change, and true selves at they play out in public spaces immortalized online.

## Supplemental Material

sj-docx-1-psp-10.1177_01461672231214629 – Supplemental material for A Tale of Two Tweets: What Factors Predict Forgiveness of Past Transgressions on Social Media? Supplemental material, sj-docx-1-psp-10.1177_01461672231214629 for A Tale of Two Tweets: What Factors Predict Forgiveness of Past Transgressions on Social Media? by Andrew J. Dawson, Sarah Williams and Anne E. Wilson in Personality and Social Psychology Bulletin

## Notes

1. Running the analyses without controlling for political leaning yields the same overall pattern of results.
