# Supplementary material for: A Tale of Two Tweets: What Factors Predict Forgiveness of Past Transgressions on Social Media?
Source: Pers Soc Psychol Bull. 2023 Dec 12;51(7):1259–80. doi: 10.1177/01461672231214629 (PMC12130597; doi:10.1177/01461672231214629)
Supplement: sj-docx-1-psp-10.1177_01461672231214629 – Supplemental material for A Tale of Two Tweets: What Factors Predict Forgiveness of Past Transgressions on Social Media? [file sj-docx-1-psp-10.1177_01461672231214629.docx]

# **A Tale of Two Tweets – Supporting Information**

The current document reports additional analyses that are not included in the main manuscript.

**Contents**

[A Tale of Two Tweets – Supporting Information 1](#_Toc148880511)

[Exclusions by Condition 1](#_Toc148880512)

[Exploratory Factor Analysis 2](#_Toc148880513)

[Aggregate Judgement Variable 2](#_Toc148880514)

[Separate Judgement Variables 3](#_Toc148880515)

[Separate Black and White Samples (Study 2) 5](#_Toc148880516)

[Tweet Evaluation 7](#_Toc148880517)

[Race of the Public Figure 7](#_Toc148880518)

[Racial Attitudes 9](#_Toc148880519)

[Table S1 11](#_Toc148880520)

[Table S2 13](#_Toc148880521)

[Table S3 15](#_Toc148880522)

[Table S4 16](#_Toc148880523)

[Table S5 19](#_Toc148880524)

[Table S6 22](#_Toc148880525)

[Table S7 24](#_Toc148880526)

[Table S8 26](#_Toc148880527)

[Table S9 27](#_Toc148880528)

[Table S10 28](#_Toc148880529)

[Figure S1 30](#_Toc148880530)

[References 32](#_Toc148880531)

# **Exclusions by Condition**

In Table S1, we report the number of participants excluded for each criterion by condition for each predictor variable.

**Alternative aggregations of judgement variable**

The authors created a series of items intended to capture different aspects of moral judgement of online transgression. In the preregistration we tentatively identified a number of conceptually separate categories (present judgement, present moral character, employer punishment, apology, resignation) that all related to the broader phenomenon. In the preregistration we did not specify how these variables would be aggregated. In the final manuscript we report two key factors (consequences and moral character judgment) informed by factor analysis (reported below). However, for full transparency, we also report in supporting information the results when all judgement items are aggregated into a single composite, and when they are separated into the five conceptual categories we mention in the preregistration. Note that factor analysis did not support splitting the scale into our five tentative conceptual categories on statistical grounds, which is why they are reported only as supplemental information.

# **Exploratory Factor Analysis**

To examine the structure of our judgement variables, we put all ten items into an exploratory factor analysis (EFA) using principal axis factoring with promax rotation as recommended by Carpenter (2018). In all three studies, the number of eigenvalues greater than 1 as well as the scree plot (see Figure S1) indicated a two-factor solution. See Table S2 for factor loadings by item. The first factor includes items related to consequences the public figure might face, including present judgement, employer firing, employer discipline, apology, and resignation while the second factor includes the four items assessing present moral character. The reverse-coded forgiveness item loaded to some degree on both factors, but unexpectedly, loadings were somewhat higher on the moral character factor although the item’s content was more aligned with the consequences factor.

To further clarify the factors, we ran confirmatory factor analyses (CFA) with the factors suggested by the EFA, as well as another CFA with the same factors but with the forgiveness item placed with the consequences variables, which was more in line with our original conceptualization of the items. Theoretically, assessment of moral character is its own construct while the consequences of how much of a person should be judged/forgiven/punished in the present would go together in their own group. Finally, we also tested the one-factor model. See Table S3 for fit statistics of each model.

Overall, the two factor models showed comparable fit scores across studies, and consistently outperformed the one-factor model, which showed poorer fit. For our main analyses reported in the main paper, we chose the model with the four-item moral character variable and six-item consequences (including forgiveness) variable. This choice was made to match the two-factor solution suggested by the eigenvalues and scree plot in the EFA while maintaining conceptual clarity by keeping forgiveness with the rest of the consequence items.

# **Aggregate Judgement Variable**

In addition to our main analyses using the two-factor model, we also conducted analyses with one factor combining the consequences and moral character items into a single variable. Overall, this aggregate judgement variable showed good reliability across studies (α = .91, .87, .93).

***Analyses***

A version of the analyses with the aggregate judgement variable is shown in Tables S4 and S5. Results are overall consistent with the main analyses.

# **Separate Judgement Variables**

Our consequences items included several different constructs, including judgement in the present, employer punishment, and ways that the public figure themselves could make amends (apology, resignation). Because these items hung together in the factor analyses and showed good reliability, we report an overall consequence variable for our analyses in the main paper (alongside the moral character variable). However, because we tentatively identified the subcomponents separately in the preregistration and they may be of interest for full transparency to examine separately, we report them separately here, first describing their measurement, then their results.

***Present Judgement***

Two items captured the degree to which the public figure should still be judged based on their past actions: “To what degree should this person be judged now based on their tweets?” and “To what degree should this person be forgiven for their tweets?”. Both items were 7-point Likert-type scales ranging from *not at all* to *very much so*. The forgiveness item was reverse-coded and the two items were aggregated into one measure of present judgment. This measure demonstrated acceptable reliability across studies, *r_SB_* = .69, .69, .77.***Employer Punishment***

Participants also rated potential consequences that could be enacted by the public figure’s employer. Two items asked about whether the public figure should be punished by his employer (one asking whether the public figure should be disciplined by his employer, and another asking whether he should be fired, from 1 (*Not at all* to 7 (*Very much so*). The two items showed good reliability, *r_SB_* = .92, .90, .94.

***Need for Apology and Resignation***

Two more items asked whether the public figure himself should take action to amend for his past transgression. On the same 7-point scale participants indicated whether the public figure should apologize for the tweets (need for apology) and whether he should resign from his current position (need for resignation). We considered aggregating these two items, but chose not to for two reasons. First, the reliability for these two items was relatively low, *r_SB_* = .58, .59, 62. Second, and more important, the two items greatly differ in extremity and participant endorsements followed descriptively different patterns (with many people endorsing need for apology for instance and only few endorsing resignation). It seemed worthwhile to report the two outcomes separately since they represent, in essence, the least severe and most severe consequence for a past transgression, and are likely to differ meaningfully.

***Analyses***

A recreation of the main analyses with the separate judgement variables is summarized in Tables S4 and S5. Overall, we find the same pattern of results as with the consequence and moral character variables. Minor differences in significance emerged for some effects in some studies, but the overall pattern was quite consistent. Participants were less harsh towards the public figure when more time had passed, and when he was younger when he posted the offensive tweets. Conservatives reacted more harshly to anti-White tweets, while liberals reacted more harshly towards anti-Black tweets.

# **Separate Black and White Samples (Study 2)**

In Study 2 we originally planned to analyse our samples of Black and White participants separately, to see if our findings held within both groups. Due to the small number of conservatives (especially in the Black sample) and low statistical power overall, we decided to use one combined sample instead. In this section we look at the samples separately.

***Black Sample***

Overall, 410 participants identified as Black. Thirty participants failed the attention check, sixty-one failed the effort check, 35 indicated in the honesty check that we should not use their data, and 85 were removed for leaning neither liberal nor conservative. The final sample included 264 participants. There was only a small minority of conservatives in this sample (*n* = 48) compared to a majority of 216 liberals.

***White Sample***

Overall, 396 participants identified as White. Fifteen participants failed the attention check, 39 failed the effort check, and 14 were removed based on the effort check. Sixty-nine participants did not identify as conservative or liberal and were removed from analyses. The final White sample after exclusions included 290 participants. Although political identity was not as unbalanced as in the Black sample, there were still over twice as many liberals (*n* = 209) as conservatives (*n* = 81).

***Analyses***

We ran the main analyses with the separate Black and White samples, and the results can be found in Tables S6 and S7. Overall, participants in White sample showed a similar (though less consistent) pattern as with the combined sample, with conservatives showing little differences across targeted race and liberals responding more harshly to anti-Black tweets. Participants in our Black sample, by contrast, typically did not show a political leaning × targeted race interaction, with both liberals and conservatives responding more harshly to anti-Black tweets. In both samples, the effects of time and age were present but inconsistent, with participants responding less harshly when more time had passed and the public figure was younger when they posted the tweets. The inconsistency in this finding across variables may be due to lower power in the separate samples.

# **Tweet Evaluation**

We also included a measure designed to assess participant’s perceptions of the tweets themselves and their moral status. Tweets were rated on a series of 7 point scales ranging from *immoral* to *moral*, *good* to *bad,*  *trivial* to *important*, *offensive* to *inoffensive*, and *funny* to *unfunny* (α = .76, .73, .78). The effects of our main independent factors on this variable are described in Table S8. Overall, this variable did not shift based on time passed or age at time of tweet, but it did show a political leaning × targeted race interaction, with liberals seeing anti-Black tweets as worse across all studies and conservatives seeing anti-White tweets as worse in Study 1 specifically.

In all three studies, for both the aggregate and separate judgement variables, we reran all of our main analyses of the judgement and subjective circumstances variables with tweet evaluation as a covariate. This was to determine whether our dependent variables could account for additional variance beyond participants initial feelings to the offensive statements themselves. These adjusted analyses did not show a consistently different pattern of results from the main analyses reported in this paper without the covariate. For more information, see syntax document on Open Science Framework.

# **Race of the Public Figure**

While the race of the public figure was intentionally kept ambiguous (by using a neutral avatar in the tweets), it occurred to us that participants may have been making assumptions on their own about his race. For this reason, in Study 3, participants were asked if they had imagined the public figure, Mike Davis, as being a particular race. The majority of participants said they did, with 85% in the anti-Black condition, 78% in the anti-White condition, and 54% in the control condition saying they had a particular race in mind.

Of those who indicated that they had made an assumption about the public figure’s race, in the anti-Black condition, almost all participants indicated that they pictured the public figure as White (97%), while in the anti-White condition, most participants indicated that they pictured the public figure as Black (90%). Although substantially fewer participants in the control condition made a guess about race, those who did generally assumed again that the public figure was White (92%).

The fact that participants were picturing a different race for the public figure in different conditions could have implications for the interpretation of our results. Judgements could be based on not just the tweets themselves, but perceptions of the *person*, whether that means attitudes towards their race itself or any assumptions based on their race. However, in the current research because the tweets themselves also contained racially offensive messages, it is likely that people’s reactions to the *message* and to the public figure’s racial *identity* would be quite conflated. While we cannot fully untangle these components in the present study, future research should address this issue more directly. Importantly, although the fact that assumptions about race differed by content condition does introduce a potential confound, our predictions would not be any different if participants were focused on the tweet or the tweeter. We would still expect liberals to give harsher judgements to White people targeting Black people and conservatives to give harsher judgements to Black people targeting White people. As a result, although further research is warranted, this complexity does not limit our ability to draw conclusions about differential partisan judgements of online offenses.

# **Racial Attitudes**

Because these studies involved racially offensive statements, we also included an exploratory measure of racial attitudes in Study 3. Participants indicated their feelings towards different groups using feelings thermometers ranging from 0° (*Very cold/Quite dislike*) to 50° (*Neutral, Neither dislike nor like*) to 100° (*Very warm/Quite like*). The items assessed attitudes towards several political and societal groups, but we were particularly interested in attitudes towards Black people and White people. It seems quite plausible that people’s attitudes toward Black people and White people could play a role in their judgments either of the racially offensive *statements* or of the public figure themselves (if they made assumptions about the person’s race). Because the focus of the current research was political orientation rather than racial attitudes, this is a secondary question; however it would be worthwhile to examine whether political leaning continues to predict judgements even after accounting for racial attitudes.

For feelings towards Black people, liberals (*M* = 79.70, *SE* = 0.96) reported higher warmth and liking than conservatives (*M* = 67.00, *SE* = 1.05), *F*(1, 1075) = 79.92, *p* < .001, η_p_^2^  = .07, but notably both groups’ liking scores were well above the neutral midpoint of 50. Conversely, for feelings towards White people, liberals (*M* = 67.83, *SE* = 0.85) reported lower warmth and liking than conservatives (*M* = 77.23, *SE* = 0.93), *F*(1, 1080) = 55.71, *p* < .001, η_p_^2^  = .05, but again, both above were well above the neutral midpoint. To ensure that our main results were not accounted for solely by differences in racial attitudes, we reran our main analyses (with all independent variables included) with the feelings thermometers for both Black and White people as simultaneous covariates. All main effects and interactions persisted while controlling for racial attitudes, though both covariates also accounted for significant variance.

For additional descriptive information, we also report the zero-order correlations between the racial thermometers and our major outcome variables split by targeted race condition in Table S10.

Overall, our findings suggest that racial attitudes do predict some variance in judgements above and beyond political leaning, but that they cannot fully account for the results found in the main paper. Likely both racial attitudes and partisanship are often related to one another, and may both play a role in people’s reactions to racist statements online.

# **Table S1**

*Number of exclusions by condition for each predictor variable.*

| **Study 1** | | | | |
| --- | --- | --- | --- | --- |
|  | 2 Years | | 7 Years | |
| Not Liberal or Conservative | 52 (12.5%) | | 48 (11.5%) | |
| Failed Attention Check(s) | 35 (8.4%) | | 44 (10.6%) | |
| Failed Honesty Check | 25 (6%) | | 19 (4.6%) | |
| Failed Bot Check | 12 (2.9%) | | 14 (3.4%) | |
| Removed for Any Reason | 99 (23.7%) | | 101 (24.3%) | |
|  |  | |  | |
|  | Age 16 | | Age 28 | |
| Not Liberal or Conservative | 51 (12.1%) | | 49 (11.9%) | |
| Failed Attention Check(s) | 46 (10.9%) | | 33 (8%) | |
| Failed Honesty Check | 22 (5.2%) | | 22 (5.4%) | |
| Failed Bot Check | 21 (5%) | | 5 (1.2%) | |
| Removed for Any Reason | 106 (25.1%) | | 94 (22.8%) | |
|  |  | |  | |
|  | Conservative | | Liberal | |
| Failed Attention Check(s) | 33 (23%) | | 29 (6%) | |
| Failed Honesty Check | 11 (4.5%) | | 20 (4.1%) | |
| Failed Bot Check | 16 (6.5%) | | 6 (1.2%) | |
| Removed for Any Reason | 51 (20.6%) | | 49 (10.1%) | |
|  |  | |  | |
|  | Anti-Black | | Anti-White | |
| Not Liberal or Conservative | 40 (9.7%) | | 60 (14.1%) | |
| Failed Attention Check(s) | 27 (6.6%) | | 52 (12.2%) | |
| Failed Honesty Check | 13 (3.1%) | | 21 (7.3%) | |
| Failed Bot Check | 1 (0.2%) | | 25 (5.9%) | |
| Removed for Any Reason | 69 (16.9%) | | 131 (30.8%) | |
|  |  | |  | |
| **Study 2** | | | | |
|  | 2 Years | | 7 Years | |
| Not Liberal or Conservative | 86 (20.7%) | | 76 (18.3%) | |
| Failed Attention Check | 28 (6.7%) | | 20 (4.8%) | |
| Failed Effort Check | 58 (13.9%) | | 49 (11.8%) | |
| Failed Honesty Check | 26 (6.3%) | | 26 (6.3%) | |
| Removed for Any Reason | 139 (33.4%) | | 124 (29.8%) | |
|  |  | |  | |
|  | Age 16 | | Age 28 | |
| Not Liberal or Conservative | 80 (19.7%) | | 82 (19.7%) | |
| Failed Attention Check | 22 (5.3%) | | 26 (6.2%) | |
| Failed Effort Check | 54 (13.0%) | | 53 (12.7%) | |
| Failed Honesty Check | 26 (6.3%) | | 26 (6.3%) | |
| Removed for Any Reason | 131 (31.6%) | | 132 (31.8%) | |
|  |  | |  | |
|  | Conservative | | Liberal | |
| Failed Attention Check | 9 (5.7%) | | 20 (4.0%) | |
| Failed Effort Check | 21 (13.2%) | | 58 (11.4%) | |
| Failed Honesty Check | 10 (6.3%) | | 28 (5.5%) | |
| Removed for Any Reason | 29 (18.2%) | | 72 (14.1%) | |
|  |  | |  | |
|  | Anti-Black | | Anti-White | |
| Not Liberal or Conservative | 78 (19.6%) | | 84 (19.1%) | |
| Failed Attention Check | 27 (6.3%) | | 21 (5.3%) | |
| Failed Effort Check | 52 (12.0%) | | 55 (13.8%) | |
| Failed Honesty Check | 22 (1.9%) | | 30 (7.5%) | |
| Removed for Any Reason | 134 (30.9%) | | 129 (32.4%) | |
|  |  | |  | |
| **Study 3** | | | | |
|  | 2 Years | | 7 Years | |
| Not Liberal or Conservative | 26 (7.5%) | | 38 (6.2%) | |
| Failed Attention Check | 29 (4.8%) | | 26 (4.2%) | |
| Failed Effort Check | 30 (4.9%) | | 26 (4.2%) | |
| Failed Honesty Check | 30 (4.8%) | | 28 (4.5%) | |
| Removed for Any Reason | 72 (11.7%) | | 64 (10.4%) | |
|  |  | |  | |
|  | Age 16 | | Age 28 | |
| Not Liberal or Conservative | 42 (6.8%) | | 42 (6.9%) | |
| Failed Attention Check | 24 (3.9%) | | 31 (5.0%) | |
| Failed Effort Check | 31 (5.0%) | | 25 (4.1%) | |
| Failed Honesty Check | 28 (4.6%) | | 30 (4.9%) | |
| Removed for Any Reason | 72 (11.7%) | | 64 (10.4%) | |
|  |  | |  | |
|  | Conservative | | Liberal | |
| Failed Attention Check | 10 (1.9%) | | 9 (1.4%) | |
| Failed Effort Check | 10 (1.9%) | | 8 (1.3%) | |
| Failed Honesty Check | 7 (1.4%) | | 12 (1.9%) | |
| Removed for Any Reason | 26 (5.0%) | | 26 (4.2%) | |
|  |  | |  | |
|  | Anti-Black | Control | | Anti-White |
| Not Liberal or Conservative | 30 (7.7%) | 34 (7.8%) | | 20 (5.0%) |
| Failed Attention Check | 17 (4.4%) | 23 (5.2%) | | 15 (3.7%) |
| Failed Effort Check | 18 (4.6%) | 25 (5.7%) | | 13 (3.2%) |
| Failed Honesty Check | 18 (4.6%) | 23 (5.3%) | | 17 (4.2%) |
| Removed for Any Reason | 47 (12.1%) | 51 (11.7%) | | 38 (9.4%) |

*Note*. Numbers in parentheses report percentage of the condition.

# **Table S2**

*Factor loadings for judgement items onto consequence and moral character factors.*

|  | Study 1 | | Study 2 | | Study 3 | |
| --- | --- | --- | --- | --- | --- | --- |
| Item | Factor 1 | Factor 2 | Factor 1 | Factor 2 | Factor 1 | Factor 2 |
| “To what degree should this person be judged now based on their tweets?” | .59 | .28 | .56 | .27 | .57 | .29 |
|  |  |  |  |  |  |  |
| “To what degree should this person be forgiven for their tweets?” (R) | .21 | .51 | .23 | .40 | .30 | .41 |
|  |  |  |  |  |  |  |
| “Should this person’s employer take disciplinary action against them, based on these tweets?” | .97 | -.11 | .91 | -.02 | .97 | -.07 |
|  |  |  |  |  |  |  |
| “Should this person’s employer fire them, based on these tweets?” | .99 | -.11 | .96 | -.05 | 1.02 | -.09 |
|  |  |  |  |  |  |  |
| “Should this person apologize for their tweets?” | .40 | .08 | .53 | -.07 | .41 | .12 |
|  |  |  |  |  |  |  |
| “Should this person resign from their job for these tweets?” | .93 | -.06 | .86 | .02 | .94 | -.03 |
|  |  |  |  |  |  |  |
| “To what degree is this specific person:” Immoral—Moral (R) | 0.04 | .73 | .00 | .73 | .07 | .80 |
|  |  |  |  |  |  |  |
| “To what degree is this specific person:” Good—Bad | -.11 | .99 | -.06 | .96 | -.01 | .92 |
|  |  |  |  |  |  |  |
| “To what degree is this specific person:” Trustworthy—Untrustworthy | -.06 | .91 | -.05 | .92 | -.07 | .97 |
|  |  |  |  |  |  |  |
| “To what degree is this specific person:” Likeable—Unlikeable | -.07 | .96 | -.05 | .94 | -.06 | .93 |

*Note*. Last four items were preceded by a preamble: “Please think about this person and give your judgements of that person in the present on the following dimensions. To what degree is this specific person:”

# **Table S3**

*Fit indices for confirmatory factor analyses by model.*

| Model | CFI | SRMR | RMSEA | χ^2^ | *df* | *p* |
| --- | --- | --- | --- | --- | --- | --- |
| Two-Factor, Forgiveness with Moral Character | .94  .95  .94 | .08  .07  .07 | .12  .11  .12 | 334.44  252.94  593.06 | 34  34  34 | < .001  < .001  < .001 |
|  |  |  |  |  |  |  |
| Two-Factor, Forgiveness with Consequences | .92  .94  .94 | .11,  .08  .08 | .14  .11  .13 | 436.04  272.07  653.64 | 34  34  34 | < .001  < .001  < .001 |
|  |  |  |  |  |  |  |
| One-Factor | .64  .67  .73 | .16  .13  .10 | .28  .26  .27 | 1862.28  1380.91  2718.75 | 35  35  35 | < .001  < .001  < .001 |

# *Note*. Indices reported for Studies 1, 2, and 3 respectively. Two-factor model consistently shows better fit than the one-factor model, and results are roughly equivalent whether forgiveness is included with moral character or consequences.**Table S4**

*Main effects and interactions with estimated marginal means and standard errors for time passed and age at time of tweet, aggregate judgement variable (all items) and separate consequence variables.*

| Study 1 | | | | | | | |
| --- | --- | --- | --- | --- | --- | --- | --- |
|  | 2 Years | | 7 Years | | Main Effect Time Passed | Main Effect  Age at Time of Tweet | Time Passed × Age at Time of Tweet |
|  | Age 16 | Age 28 | Age 16 | Age 28 |  |  |  |
| Aggregate Judgement | 4.30  (0.11) | 4.63  (0.11) | 3.99  (0.11) | 4.16  (0.11) | *F*(1, 618) = 13.23,  h_p_^2^ = .02*** [.01, .05] | *F*(1, 618) = 5.54,  h_p_^2^ = .01* [< .001, .03] | *F*(1, 618) = 0.58,  h_p_^2^ = .001 [< .001, .01] |
|  |  |  |  |  |  |  |  |
| Present Judgement | 4.19  (0.13) | 4.59  (0.130 | 3.76  (0.13) | 4.00  (0.13) | *F*(1, 618) = 16.07,  η_p_^2^ = .03*** [.01, .05] | *F*(1, 618) = 6.13,  η_p_^2^ = .01* [< .001, .03] | *F*(1, 618) = 0.40,  η_p_^2^ = .001 [< .001, .01] |
|  |  |  |  |  |  |  |  |
| Employer Punishment | 3.82  (0.17) | 4.25  (0.18) | 3.58  (0.17) | 3.65  (0.18) | *F*(1, 618) = 5.95,  η_p_^2^ = .01* [< .001, .03] | *F*(1, 618) = 2.13,  η_p_^2^ = .003 [< .001, .02] | *F*(1, 618) = 1.04,  η_p_^2^ = .002 [< .001, .01] |
|  |  |  |  |  |  |  |  |
| Apology | 5.62  (0.15) | 5.67  (0.16) | 5.59  (0.16) | 5.49  (0.16) | *F*(1, 618) = 0.46,  η_p_^2^ = .001 [< .001, .01] | *F*(1, 618) = 0.02,  η_p_^2^ < .001 [< .001, .01] | *F*(1, 618) = 0.27,  η_p_^2^ < .001 [< .001, .01] |
|  |  |  |  |  |  |  |  |
| Resignation | 3.51  (0.17) | 3.72  (0.18) | 3.14  (0.18) | 3.12  (0.18) | *F*(1, 615) = 7.55,  η_p_^2^ = .01** [< .001, .03] | *F*(1, 615) = 0.29,  η_p_^2^ < .001 [< .001, .01] | *F*(1, 615) = 0.47,  η_p_^2^ = .001 [< .001, .01] |
| Study 2 | | | | | | | |
|  | 2 Years | | 7 Years | | Main Effect Time Passed | Main Effect  Age at Time of Tweet | Time Passed × Age at Time of Tweet |
|  | Age 16 | Age 28 | Age 16 | Age 28 |  |  |  |
| Aggregate Judgement | 4.41  (0.11) | 4.69  (0.11) | 4.13  (0.10) | 4.52  (0.10) | *F*(1, 552) = 4.28,  η_p_^2^ = .01* [< .001, .03] | *F*(1, 552) = 9.77,  η_p_^2^ = .02** [.002, .04] | *F*(1, 552) = 0.29,  η_p_^2^ = .001 [< .001, .01] |
|  |  |  |  |  |  |  |  |
| Present Judgement | 3.82  (0.15) | 4.30  (0.14) | 3.29  (0.13) | 3.84  (0.13) | *F*(1, 552) = 13.19,  η_p_^2^ = .02*** [< .001, .05] | *F*(1, 552) = 13.99,  η_p_^2^ = .03*** [.01, .06] | *F*(1, 552) = 0.07  η_p_^2^ < .001 [< .001, .01] |
|  |  |  |  |  |  |  |  |
| Employer Punishment | 2.97  (0.20) | 3.88  (0.19) | 2.72  (0.18) | 3.29  (0.17) | *F*(1, 552) = 5.07,  η_p_^2^ = .01* [< .001, .03] | *F*(1, 552) = 15.91,  η_p_^2^ = .03*** [.01, .06] | *F*(1, 552) = 0.83,  η_p_^2^ = .002 [< .001, .01] |
|  |  |  |  |  |  |  |  |
| Apology | 5.37  (0.19) | 5.40  (0.19) | 5.22  (0.18) | 5.50  (0.17) | *F*(1, 550) = 0.02,  η_p_^2^ < .001 [< .001, .01] | *F*(1, 550) = 0.71,  η_p_^2^ = .001 [< .001, .01] | *F*(1, 550) = 0.45,  η_p_^2^ = .001 [< .001, .01] |
|  |  |  |  |  |  |  |  |
| Resignation | 2.74  (0.19) | 3.25  (0.19) | 2.27  (0.18) | 2.63  (0.17) | *F*(1, 551) = 8.72,  η_p_^2^ = .02** [< .001, .04] | *F*(1, 551) = 5.65,  η_p_^2^ = .01* [< .001, .03] | *F*(1, 551) = 0.18,  η_p_^2^ < .001 [< .001, .01] |
| Study 3 | | | | | | | |
|  | 2 Years | | 7 Years | | Main Effect Time Passed | Main Effect  Age at Time of Tweet | Time Passed × Age at Time of Tweet |
|  | Age 16 | Age 28 | Age 16 | Age 28 |  |  |  |
| Aggregate Judgement | 4.31  (0.08) | 4.70  (0.08) | 3.71  (0.08) | 4.33  (0.08) | *F*(1, 1070) = 40.21,  η_p_^2^ = .04*** [.02, .06] | *F*(1, 1070) = 45.01,  η_p_^2^ = .04*** [.02, .07] | *F*(1, 1070) = 2.49,  η_p_^2^ = .002 [< .001, .01] |
|  |  |  |  |  |  |  |  |
| Present Judgement | 4.06  (0.09) | 4.54  (0.09) | 3.40  (0.09) | 4.10  (0.09) | *F*(1, 1070) = 36.36,  η_p_^2^ = .03*** [.02, .06] | *F*(1, 1070) = 41.07,  η_p_^2^ = .04*** [.02, .06] | *F*(1, 1070) = 1.36,  η_p_^2^ = .001 [< .001, .01] |
|  |  |  |  |  |  |  |  |
| Employer Punishment | 3.40  (0.12) | 4.07  (0.11) | 2.71  (0.11) | 3.51  (0.11) | *F*(1, 1069) = 30.02,  η_p_^2^ = .03*** [.01, .05] | *F*(1, 1069) = 41.25,  η_p_^2^ = .04*** [.02, .06] | *F*(1, 1069) = 0.40,  η_p_^2^ < .001 [< .001, .01] |
|  |  |  |  |  |  |  |  |
| Apology | 5.93  (0.10) | 5.94  (0.10) | 5.70  (0.10) | 5.88  (0.10) | *F*(1, 1068) = 2.04,  η_p_^2^ = .002 [< .001, .01] | *F*(1, 1068) = 0.88,  η_p_^2^ = .001 [< .001, .01] | *F*(1, 1068) = 0.71,  η_p_^2^ = .001 [< .001, .01] |
|  |  |  |  |  |  |  |  |
| Resignation | 2.97  (0.12) | 3.53  (0.12) | 2.43  (0.12) | 2.98  (0.12) | *F*(1, 1070) = 21.79,  η_p_^2^ = .02*** [.01, .04] | *F*(1, 1070) = 22.27,  η_p_^2^ = .02*** [.01, .04] | *F*(1, 1070) = 0.001,  η_p_^2^ < .001 [< .001, < .001] |

*Note*. †*p* < .10, * *p* < .05, ** *p* < .01, *** *p* < .001

# **Table S5**

*Main effects and interactions with estimated marginal means and standard errors for political leaning and targeted race, aggregate judgement variable and separate consequence variables.*

| Study 1 | | | | | | | | | | | | | | | |
| --- | --- | --- | --- | --- | --- | --- | --- | --- | --- | --- | --- | --- | --- | --- | --- |
|  | | Conservative | | | | Liberal | | | | Main Effect Time Political Leaning | | Main Effect  Targeted Race | | Political Leaning × Targeted Race | |
|  | | Anti-Black | | Anti-White | | Anti-Black | | Anti-White | |  | |  | |  | |
| Aggregate  Judgement | | 4.07_Aa_  (0.13) | | 4.40A_a_  (0.13) | | 4.63_ab_  (0.09) | | 3.98_bb_  (0.08) | | *F*(1, 618) = 0.41,  η_p_^2^ = .001 [< .001, .01] | | *F*(1, 618) = 2.07,  η_p_^2^ = .003 [< .001, .02] | | *F*(1, 618) = 20.23,  η_p_^2^ = .03*** [.01, .06] | |
|  | |  | |  | |  | |  | |  | |  | |  | |
| Present Judgement | | 3.85_aa_  (0.15) | | 4.34_ba_  (0.15) | | 4.49_ab_  (0.11) | | 3.86_bb_  (0.10) | | *F*(1, 618) = 0.40,  η_p_^2^ = .001 [< .001, .01] | | *F*(1, 618) = 0.30,  η_p_^2^ < .001 [< .001, .01] | | *F*(1, 618) = 18.84,  η_p_^2^ = .03*** [.01, .06] | |
|  | |  | |  | |  | |  | |  | |  | |  | |
| Employer Punishment | | 3.66_Aa_  (0.20) | | 4.17_AA_  (0.21) | | 4.10_ab_  (0.14) | | 3.38_bA_  (0.13) | | *F*(1, 618) = 1.00,  η_p_^2^ = .002 [< .001, .01] | | *F*(1, 618) = 0.36,  η_p_^2^ = .001 [< .001, .01] | | *F*(1, 618) = 12.55,  η_p_^2^ = .02*** [< .001, .05] | |
|  | |  | |  | |  | |  | |  | |  | |  | |
| Apology | | 5.24_aa_  (0.18) | | 5.62_aa_  (0.18) | | 4.99_ab_  (0.13) | | 5.51_ba_  (0.12) | | *F*(1, 618) = 4.32,  η_p_^2^ = .01* [< .001, .03] | | *F*(1, 618) = 0.10,  η_p_^2^ < .001 [< .001, .01] | | *F*(1, 618) = 7.74,  η_p_^2^ = .01** [< .001, .04] | |
|  | |  | |  | |  | |  | |  | |  | |  | |
| Resignation | | 3.34_aA_  (0.21) | | 3.61_aa_  (0.21) | | 3.80_aA_  (0.15) | | 2.75_bb_  (0.13) | | *F*(1, 615) = 1.29,  η_p_^2^ = .002 [< .001, .02] | | *F*(1, 615) = 4.97,  η_p_^2^ = .01* [< .001, .03] | | *F*(1, 615) = 13.90,  η_p_^2^ = .02*** [< .001, .05] | |
| Study 2 | | | | | | | | | | | | | | | |
|  | | Conservative | | | | Liberal | | | | Main Effect Time Political Leaning | | Main Effect  Targeted Race | | Political Leaning × Targeted Race | |
|  | | Anti-Black | | Anti-White | | Anti-Black | | Anti-White | |  | |  | |  | |
| Aggregate  Judgement | | 4.29_aa_  (0.14) | | 4.33_aA_  (0.13) | | 5.07_ab_  (0.07) | | 4.06_bA_  (0.07) | | *F*(1, 552) = 5.58,  η_p_^2^ = .01* [< .001, .03] | | *F*(1, 552) = 21.04,  η_p_^2^ = .04*** [.01, .07] | | *F*(1, 552) = 24.86,  η_p_^2^ = .04*** [.02, .08] | |
|  | |  | |  | |  | |  | |  | |  | |  | |
| Present Judgement | | 3.68_aa_  (0.18) | | 3.68_aa_  (0.17) | | 4.44_ab_  (0.10) | | 3.45_ba_  (0.09) | | *F*(1, 552) = 3.87,  η_p_^2^ = .01* [< .001, .03] | | *F*(1, 552) = 13.09,  η_p_^2^ = .02*** [< .001, .05] | | *F*(1, 552) = 12.65  η_p_^2^ = .02*** [< .001, .05] | |
|  | |  | |  | |  | |  | |  | |  | |  | |
| Employer Punishment | | 3.10_aa_  (0.24) | | 3.12_aa_  (0.22) | | 3.95_ab_  (0.13) | | 2.71_ba_  (0.12) | | *F*(1, 552) = 1.42,  η_p_^2^ = .003 [< .001, .02] | | *F*(1, 552) = 10.79  η_p_^2^ = .02** [< .001, .05] | | *F*(1, 552) = 11.52  η_p_^2^ = .02*** [< .001, .05] | |
|  | |  | |  | |  | |  | |  | |  | |  | |
| Apology | | 4.93_aa_  (0.23) | | 5.21_aa_  (0.22) | | 6.14_ab_  (0.13) | | 5.20_ba_  (0.12) | | *F*(1, 550) = 10.76,  η_p_^2^ = .02** [< .001, .05] | | *F*(1, 550) = 3.15,  η_p_^2^ = .01† [< .001, .02] | | *F*(1, 550) = 11.16,  η_p_^2^ = .02*** [< .001, .05] | |
|  | |  | |  | |  | |  | |  | |  | |  | |
| Resignation | | 2.71_aa_  (0.23) | | 2.67_aA_  (0.22) | | 3.34_ab_  (0.13) | | 2.17_bA_  (0.12) | | *F*(1, 551) = 0.14,  η_p_^2^ < .001 [< .001, .01] | | *F*(1, 551) = 10.93,  η_p_^2^ = .02** [< .001, .05] | | *F*(1, 551) = 9.36,  η_p_^2^ = .02** [< .001, .04] | |
| Study 3 | | | | | | | | | | | | | | | |
|  | Conservative | | | | | Liberal | | | | | Main Effect Time Political Leaning | | Main Effect  Targeted Race | | Political Leaning × Targeted Race |
|  | Anti-Black | | Control | | Anti-White | Anti-Black | Control | | Anti-White | |  |  | |  | |
| Aggregate  Judgement | 3.91_aa_  (0.10) | | 3.41_ba_  (0.10) | | 4.33_ca_  (0.10) | 5.33_ab_  (0.10) | 4.28_bb_  (0.08) | | 4.32_ba_  (0.09) | | *F*(1, 1070) = 99.57,  η_p_^2^ = .09*** [.06, .12] | *F*(2, 1070) = 36.09,  η_p_^2^ = .06*** [.04, .09] | | *F*(2, 1070) = 29.66,  v_p_^2^ = .05*** [.03, .08] | |
|  |  | |  | |  |  |  | |  | |  |  | |  | |
| Present Judgement | 3.67_aa_  (0.11) | | 3.15_ba_  (0.12) | | 4.13_ca_  (0.12) | 5.06_ab_  (0.12) | 4.07_bb_  (0.10) | | 4.07_ba_  (0.10) | | *F*(1, 1070) = 66.24,  η_p_^2^ = .06*** [.03, .09] | *F*(2, 1070) = 23.59,  η_p_^2^ = .04*** [.02, .07] | | *F*(2, 1070) = 21.51,  η_p_^2^ = .04*** [.02, .06] | |
|  |  | |  | |  |  |  | |  | |  |  | |  | |
| Employer Punishment | 2.95_aa_  (0.14) | | 2.23_ba_  (0.15) | | 3.47_ca_  (0.15) | 4.94_ab_  (0.14) | 3.31_Bb_  (0.13) | | 3.63_Ba_  (0.13) | | *F*(1, 1069) = 88.86,  η_p_^2^ = .08*** [.05, .11] | *F*(2, 1069) = 37.27,  η_p_^2^ = .07*** [.04, .09] | | *F*(2, 1069) = 20.80,  η_p_^2^ = .04*** [.02, .06] | |
|  |  | |  | |  |  |  | |  | |  |  | |  | |
| Apology | 5.61_aa_  (0.13) | | 4.95_ba_  (0.13) | | 5.68_aa_  (0.13) | 6.74_ab_  (0.13) | 6.08_bb_  (0.11) | | 6.11_bb_  (0.11) | | *F*(1, 1068) = 80.00,  η_p_^2^ = .07*** [.04, .10] | *F*(2, 1068) = 14.53,  η_p_^2^ = .03*** [.01, .05] | | *F*(2, 1068) = 5.51,  η_p_^2^ = .01** [< .001, .02] | |
|  |  | |  | |  |  |  | |  | |  |  | |  | |
| Resignation | 2.49_aa_  (0.15) | | 1.85_ba_  (0.15) | | 3.13_ca_  (0.16) | 4.57_ab_  (0.15) | 2.82_bb_  (0.13) | | 3.00_ba_  (0.13) | | *F*(1, 1070) = 68.49,  η_p_^2^ = .06*** [.04, .09] | *F*(2, 1070) = 35.27,  η_p_^2^ = .06*** [.04, .09] | | *F*(2, 1070) = 28.58,  η_p_^2^ = .05*** [.03, .08] | |

*Note*. Different subscript letters across means denotes a significant difference. First subscript refers to simple effects of targeted race with both conservatives and liberals. Second subscript refers to simple effects of political leaning for anti-Black and anti-White tweets. Marginal differences are denoted by the same letter in all caps. †p < .10, * p < .05, ** p < .01, *** p < .001

# **Table S6**

*Main effects and interactions with estimated marginal means and standard errors for time passed and age at time of tweet, separate Black and White samples (Study 2).*

| Black Sample | | | | | | | |
| --- | --- | --- | --- | --- | --- | --- | --- |
|  | 2 Years | | 7 Years | | Main Effect Time Passed | Main Effect  Age at Time of Tweet | Time Passed × Age at Time of Tweet |
|  | Age 16 | Age 28 | Age 16 | Age 28 |  |  |  |
| Time Memory Check | 2.69  (0.42) | 2.11  (0.30) | 6.14  (0.27) | 6.04  (0.26) | *F*(1, 246) = 132.99,  η_p_^2^ = .35*** [.26, .42] | *F*(1, 246) = 1.11,  η_p_^2^ = .01 [< .001, .04] | *F*(1, 246) = 0.54,  η_p_^2^ = .002 [<.001, .03] |
|  |  |  |  |  |  |  |  |
| Age Memory Check | 16.78  (0.75) | 26.89  (0.58) | 17.45  (0.48) | 26.41  (0.47) | *F*(1, 245) = 0.03,  η_p_^2^ < .001 [<.001, .01] | *F*(1, 245) = 272.32,  η_p_^2^ = .53*** [0.45, 0.59] | *F*(1, 245) = 1.00,  η_p_^2^ = .004 [< .001, .03] |
|  |  |  |  |  |  |  |  |
| Subjective Time | 31.04  (6.90) | 33.62  (4.90) | 46.08  (4.39) | 43.51  (4.29) | *F*(1, 248) = 5.68,  η_p_^2^ = .02* [<.001, .07] | *F*(1, 248) < .001,  η_p_^2^ < .001 [< .001, < .001] | *F*(1, 248) = 0.24,  η_p_^2^ = .001 [< .001, .02] |
|  |  |  |  |  |  |  |  |
| Subjective Age | 4.10  (0.31) | 5.81  (0.22) | 3.76  (0.19) | 5.17  (0.19) | *F*(1, 248) = 4.43,  η_p_^2^ = .02* [<.001, .06] | *F*(1, 248) = 45.43,  η_p_^2^ = .16*** [.08, .24] | *F*(1, 248) = 0.42,  η_p_^2^ = .002 [< .001, .03] |
|  |  |  |  |  |  |  |  |
| Consequences | 4.18  (0.31) | 4.27  (0.22) | 3.59  (0.19) | 4.14  (0.19) | *F*(1, 248) = 2.46,  η_p_^2^ = .003 [< .001, .03] | *F*(1, 248) = 1.93,  η_p_^2^ = .01 [< .001, .05] | *F*(1, 248) = 0.98,  η_p_^2^ = .004 [< .001, .03] |
|  |  |  |  |  |  |  |  |
| Moral Character | 4.78  (0.29) | 4.55  (0.21) | 4.35  (0.18) | 4.51  (0.18) | *F*(1, 245) = 1.12,  η_p_^2^ = .004 [< .001, .04] | *F*(1, 245) = 0.02,  η_p_^2^ < .001 [< .001, .01] | *F*(1, 245) = 0.80,  η_p_^2^ = .003 [< .001, .03] |
| White Sample | | | | | | | |
|  | 2 Years | | 7 Years | | Main Effect Time Passed | Main Effect  Age at Time of Tweet | Time Passed × Age at Time of Tweet |
|  | Age 16 | Age 28 | Age 16 | Age 28 |  |  |  |
| Time Memory Check | 2.22  (0.18) | 2.51  (0.20) | 6.55  (0.18) | 6.45  (0.19) | *F*(1, 274) = 492.70,  η_p_^2^ = .64*** [.58, .69] | *F*(1, 274) = 0.24,  η_p_^2^ = .001 [< .001, .02] | *F*(1, 274) = 1.06,  η_p_^2^ = .004 [< .001, .03] |
|  |  |  |  |  |  |  |  |
| Age Memory Check | 16.71  (0.32) | 26.29  (0.36) | 16.72  (0.33) | 26.24  (0.34) | *F*(1, 271) = 0.01,  η_p_^2^ < .001 [< .001. .01] | *F*(1, 271) = 809.43,  η_p_^2^ = .75*** [.70, .79] | *F*(1, 271) = 0.01,  η_p_^2^ < .001 [< .001, .01] |
|  |  |  |  |  |  |  |  |
| Subjective Time | 44.12  (3.29) | 38.68  (3.60) | 70.23  (3.28) | 54.79  (3.37) | *F*(1, 273) = 38.90,  η_p_^2^ = .13*** [.06, .20] | *F*(1, 273) = 9.52,  η_p_^2^ = .03** [< .001, .09] | *F*(1, 273) = 2.18,  η_p_^2^ = .01 [< .001, .04] |
|  |  |  |  |  |  |  |  |
| Subjective Age | 3.75  (0.15) | 5.35  (0.16) | 3.42  (0.15) | 5.17  (0.15) | *F*(1, 274) = 2.81,  η_p_^2^ = .01† [< .001, .05] | *F*(1, 274) = 120.89,  η_p_^2^ = .31*** [.22, .39] | *F*(1, 274) = 0.22,  η_p_^2^ = .001 [< .001, .02] |
|  |  |  |  |  |  |  |  |
| Consequences | 3.46  (0.17) | 4.06  (0.19) | 2.98  (0.18) | 3.37  (0.18) | *F*(1, 274) = 10.62,  η_p_^2^ = .04** [.01, .09] | *F*(1, 274) = 7.79,  η_p_^2^ = .03** [< .001, .08] | *F*(1, 274) = 0.33,  η_p_^2^ = .001 [< .001, .02] |
|  |  |  |  |  |  |  |  |
| Moral Character | 4.19  (0.14) | 4.48  (0.15) | 3.87  (0.14) | 4.20  (0.14) | *F*(1, 273) = 4.34,  η_p_^2^ = .02* [< .001, .06] | *F*(1, 273) = 4.92,  η_p_^2^ = .02* [< .001, .06] | *F*(1, 273) = 0.03,  η_p_^2^ < .001 [< .001, .01] |

*Note*. †*p* < .10, * *p* < .05, ** *p* < .01, *** *p* < .001

# **Table S7**

*Main effects and interactions with estimated marginal means and standard errors for political leaning and targeted race, separate Black and White samples (Study 2).*

| Black Sample | | | | | | | |
| --- | --- | --- | --- | --- | --- | --- | --- |
|  | Conservative | | Liberal | | Main Effect Political Leaning | Main Effect  Targeted Race | Political Leaning × Targeted Race |
|  | Anti-Black (*N* = 24) | Anti-White  (*N* = 24) | Anti-Black  (*N* = 101) | Anti-White  (*N* = 115) |  |  |  |
| Consequences | 4.58_Aa_  (0.32) | 3.74_AA_  (0.28) | 4.73_aa_  (0.13) | 3.14_bA_  (0.12) | *F*(1, 248) = 0.93,  η_p_^2^ = .003 [< .001, .03] | *F*(1, 248) = 27.74,  η_p_^2^ = .10*** [.04, .18] | *F*(1, 248) = 2.69,  η_p_^2^ = .01 [< .001, .05] |
|  |  |  |  |  |  |  |  |
| Moral Character | 5.05_aa_  (0.30) | 4.11_ba_  (0.27) | 5.06_aa_  (0.12) | 3.96_ba_  (0.12) | *F*(1, 245) = 0.11,  η_p_^2^ < .001 [< .001, .02] | *F*(1, 245) = 21.44,  η_p_^2^ = .08*** [.03, .15] | *F*(1, 245) = 0.13,  η_p_^2^ < .001 [< .001, .02] |
|  |  |  |  |  |  |  |  |
| Current Relevance | 5.08_aa_  (0.29) | 3.88_ba_  (0.26) | 5.11_aa_  (0.12) | 3.86_bb_  (0.11) | *F*(1, 248) = 0.001,  η_p_^2^ < .001 [< .001. < .001] | *F*(1, 248) = 33.58,  η_p_^2^ = .12*** [.05, .20] | *F*(1, 248) = 0.01,  η_p_^2^ < .001 [< .001, .01] |
|  |  |  |  |  |  |  |  |
| Statute of Limitations | 160.02_Aa_  (46.82) | 56.60_Aa_  (41.39) | 128.53_aa_  (19.23) | 88.18_aa_  (19.04) | *F*(1, 236) < .001,  η_p_^2^ < .001 [<.001, < .001] | *F*(1, 236) = 4.49,  η_p_^2^ = .02* [<.001, .07] | *F*(1, 236) = 0.86,  η_p_^2^ = .004 [< .001, .03] |
|  |  |  |  |  |  |  |  |
| Subjective Time | 30.48_aa_  (7.23) | 38.82_aA_  (6.42) | 34.61_aa_  (2.91) | 50.34_bA_  (2.72) | *F*(1, 248) = 2.24,  η_p_^2^ = .01* [< .001, .05] | *F*(1, 248) = 5.30,  η_p_^2^ = .02* [< .001, .07] | *F*(1, 248) = 0.50,  η_p_^2^ = .002 [< .001, .03] |
|  |  |  |  |  |  |  |  |
| Subjective Age | 5.07_aa_  (0.32) | 4.14_ba_  (0.28) | 5.12_aa_  (0.13) | 4.51_ba_  (0.12) | *F*(1, 248) = 0.79,  η_p_^2^ = .003 [< .001, .03] | *F*(1, 248) = 11.16,  η_p_^2^ = .04*** [.01, .10] | *F*(1, 248) = 0.48,  η_p_^2^ = .002 [< .001, .03] |
| White Sample | | | | | | | |
|  | Conservative | | Liberal | | Main Effect Political Leaning | Main Effect  Targeted Race | Political Leaning × Targeted Race |
|  | Anti-Black  (*N* = 38) | Anti-White  (*N* = 43) | Anti-Black  (*N* = 98) | Anti-White  (*N* = 111) |  |  |  |
| Consequences | 3.02_aa_  (0.22) | 3.46_aa_  (0.21) | 3.44_ab_  (0.13) | 3.96_ba_  (0.14) | *F*(1, 274) = 6.63,  η_p_^2^ = .02* [< .001, .07] | *F*(1, 274) = 0.04,  η_p_^2^ < .001 [< .001, .01] | *F*(1, 274) = 7.11,  η_p_^2^ .03** [< .001, .07] |
|  |  |  |  |  |  |  |  |
| Moral Character | 3.72_aa_  (0.17) | 4.32_ba_  (0.16) | 4.54_ab_  (0.11) | 4.15_ba_  (0.10) | *F*(1, 273) = 5.23,  η_p_^2^ = .02* [< .001, .06] | *F*(1, 273) = 0.56,  η_p_^2^ = .002 [< .001, .03] | *F*(1, 273) = 12.20,  η_p_^2^ = .04*** [.01, .10] |
|  |  |  |  |  |  |  |  |
| Current Relevance | 3.71_aa_  (0.19) | 4.14_ba_  (0.18) | 4.54_ab_  (0.12) | 4.12_ba_  (0.11) | *F*(1, 274) = 6.95,  η_p_^2^ = .03** [< .001, .07] | *F*(1, 274) = 0.001,  η_p_^2^ < .001 [< .001. < .001] | *F*(1, 274) = 7.51,  η_p_^2^ = .03** [< .001, .07] |
|  |  |  |  |  |  |  |  |
| Statute of Limitations | 54.21_aa_  (22.44) | 83.56_aa_  (21.86) | 121.55_Ab_  (14.15) | 85.90_Aa_  (13.64) | *F*(1, 260) = 3.55,  η_p_^2^ = .01† [< .001, .05] | *F*(1, 260) = 0.03  η_p_^2^ < .001 [< .001, .01] | *F*(1, 260) = 3.09,  η_p_^2^ = .01† [< .001, .05] |
|  |  |  |  |  |  |  |  |
| Subjective Time | 60.49_Aa_  (4.19) | 50.08_Aa_  (3.91) | 43.83_ab_  (2.60) | 53.42_ba_  (2.49) | *F*(1, 273) = 3.87,  η_p_^2^ = .01* [< .001, .05] | *F*(1, 273) = 0.02,  η_p_^2^ < .001 [< .001, .01] | *F*(1, 273) = 8.72,  η_p_^2^ = .03** [< .001, .08] |
|  |  |  |  |  |  |  |  |
| Subjective Age | 4.32_aa_  (0.19) | 4.47_aA_  (0.18) | 4.77_ab_  (0.12) | 4.12_bA_  (0.11) | *F*(1, 274) = 0.10,  η_p_^2^ < .001 [< .001, .02] | *F*(1, 274) = 2.65,  η_p_^2^ = .01 [< .001, .04] | *F*(1, 274) = 6.84,  η_p_^2^ = .02** [< .001, .07] |

*Note*. Different subscript letters across means denotes a significant difference. First subscript refers to simple effects of targeted race with both conservatives and liberals. Second subscript refers to simple effects of political leaning for anti-Black and anti-White tweets. Marginal differences are denoted by the same letter in all caps. †p < .10, * p < .05, ** p < .01, *** p < .001

# **Table S8**

*Main effects and interactions with estimated marginal means and standard errors for tweet evaluation variable.*

|  | 2 Years | | | | 7 Years | | | | Main Effect Time Passed | Main Effect  Age at Time of Tweet | Time Passed × Age at Time of Tweet |
| --- | --- | --- | --- | --- | --- | --- | --- | --- | --- | --- | --- |
|  | Age 16 | | Age 28 | | Age 16 | | Age 28 | |  |  |  |
| Study 1 | 5.60  (0.09) | | 5.51  (0.10) | | 5.42  (0.10) | | 5.47  (0.10) | | *F*(1, 618) = 1.28,  η_p_^2^ = .002 [< .001, .02] | *F*(1, 618) = 0.07,  η_p_^2^ < .001 [< .001, .01] | *F*(1, 618) = 0.51,  η_p_^2^ = .001 [< .001, .01] |
|  |  | |  | |  | |  | |  |  |  |
| Study 2 | 5.75  (0.11) | | 5.55  (010) | | 5.72  (0.10) | | 5.72  (0.09) | | *F*(1, 551) = 0.42,  η_p_^2^ = .001 [< .001, .01] | *F*(1, 551) =1.03,  η_p_^2^ = .002 [< .001, .02] | *F*(1, 551) = 1.03,  η_p_^2^ = .002 [< .001, 0.02] |
|  |  | |  | |  | |  | |  |  |  |
| Study 3 | 5.97  (0.06) | | 5.88  (0.06) | | 5.96  (0.06) | | 5.92  (0.06) | | *F*(1, 1070) = 0.07,  η_p_^2^ < .001 [< .001, .01] | *F*(1, 1070) = 1.29,  η_p_^2^ = .001 [< .001, < .001] | *F*(1, 1070) = 0.15,  η_p_^2^ < .001 [< .001, < .001] |
|  | Conservative | | | | Liberal | | | | Main Effect Time Political Leaning | Main Effect  Targeted Race | Political Leaning × Targeted Race |
|  | Anti-Black | Control | | Anti-White | Anti-Black | Control | | Anti-White |  |  |  |
| Study 1 | 5.10_aa_  (0.11) | — | | 5.47_ba_  (0.12) | 5.92_ab_  (0.08) | — | | 5.51_ba_  (0.07) | *F*(1, 618) = 19.50,  η_p_^2^ = .03*** [.01, .06] | *F*(1, 618) = 0.04,  η_p_^2^ < .002 [< .001, .01] | *F*(1, 618) = 16.07,  η_p_^2^ = .03*** [.01, .05] |
|  |  |  | |  |  |  | |  |  |  |  |
| Study 2 | 5.59_aa_  (0.13) | — | | 5.61_aa_  (0.12) | 6.15_ab_  (0.07) | — | | 5.39_ba_  (0.07) | *F*(1, 551) = 2.75,  η_p_^2^ = .01† [< .001, .02] | *F*(1, 551) = 13.49,  η_p_^2^ = .02*** [.01, .05] | *F*(1, 551) = 15.31,  η_p_^2^ = .03*** [.01, .06] |
|  |  |  | |  |  |  | |  |  |  |  |
| Study 3 | 5.89_aa_  (0.07) | 5.52_ba_  (0.07) | | 6.03_aa_  (0.08) | 6.46_ab_  (0.07) | 5.89_bb_  (0.06) | | 5.81_bb_  (0.07) | *F*(1, 1070) = 17.22,  η_p_^2^ = .02*** [< .001, .03] | *F*(2, 1070) = 22.64,  η_p_^2^ = .04*** [.02, .07] | *F*(2, 1070) = 16.89,  η_p_^2^ = .03*** [.01, .05] |

*Note*. Different subscript letters across means denotes a significant difference. First subscript refers to simple effects of targeted race with both conservatives and liberals. Second subscript refers to simple effects of political leaning for anti-Black and anti-White tweets. Marginal differences are denoted by the same letter in all caps. †p < .10, * p < .05, ** p < .01, *** p < .001

# **Table S9**

*Chi-square analyses with estimated marginal means and standard errors for the categorical true self variable in Study 3.*

|  | Conservatives  χ^2^ (2) = 4.97† | | | Liberals  χ^2^ (2) = 8.36* | | |
| --- | --- | --- | --- | --- | --- | --- |
|  | Anti-Black | Anti-White | Control | Anti-Black | Anti-White | Control |
| True Self | 39.13_a_ | 45.52_a_ | 32.89_a_ | 56.71_a_ | 41.45_b_ | 47.14_a,b_ |
| Surface Self | 60.87_a_ | 54.48_a_ | 67.11_a_ | 43.29_a_ | 58.55_b_ | 52.86_a,b_ |

*Note*. Chi-square values indicate association between targeted race and true self, †p < .10, * p < .05. Percentages are the proportion of participants who chose the given option (true self or surface self) within the targeted race condition. Different subscript letters across percentages denotes a significant difference (but not the direction of the difference) across conditions of targeted race. Cells with both a and b do not differ from cells containing either letter.

# **Table S10**

*Correlations between political leaning, racial attitudes, and outcome variables, split by targeted race condition.*

| **Anti-Black Condition** | | | | | |
| --- | --- | --- | --- | --- | --- |
|  | 1 | 2 | 3 | 4 | 5 |
| 1. Attitudes Black People | — | — | — | — | — |
| 2. Attitudes White People | .32*** | — | — | — | — |
| 3. Political Leaning | .31*** | -.17*** | — | — | — |
| 4. Consequences | .39*** | -.12* | .50*** | — | — |
| 5. Moral Character | .35*** | -.18*** | .40*** | .73*** | — |
| **Anti-White Condition** | | | | | |
|  | 1 | 2 | 3 | 4 | 5 |
| 1. Attitudes Black People | — | — | — | — | — |
| 2. Attitudes White People | .39*** | — | — | — | — |
| 3. Political Leaning | .16** | -.27*** | — | — | — |
| 4. Consequences | -.09† | .08 | .01 | — | — |
| 5. Moral Character | -.15** | .03 | -.08 | .65*** | — |
| **Control Condition** | | | | | |
|  | 1 | 2 | 3 | 4 | 5 |
| 1. Attitudes Black People | — | — | — | — | — |
| 2. Attitudes White People | .33*** | — | — | — | — |
| 3. Political Leaning | .32*** | -.22*** | — | — | — |
| 4. Consequences | .22*** | -.17*** | .36*** | — | — |
| 5. Moral Character | .06 | -.19*** | .26*** | .70*** | — |

*Note*. Higher scores for racial attitudes measures mean more positive feelings towards group. Political leaning was coded conservative = 0, liberal = 1. †p < .10, * p < .05, ** p < .01, *** p < .001

# **Figure S1**

*Scree plots for exploratory factor analysis, consequence and moral character items*

**Study 1**


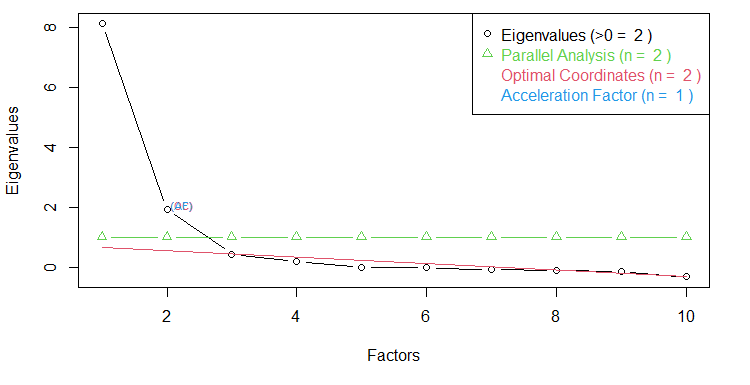


**Study 2**


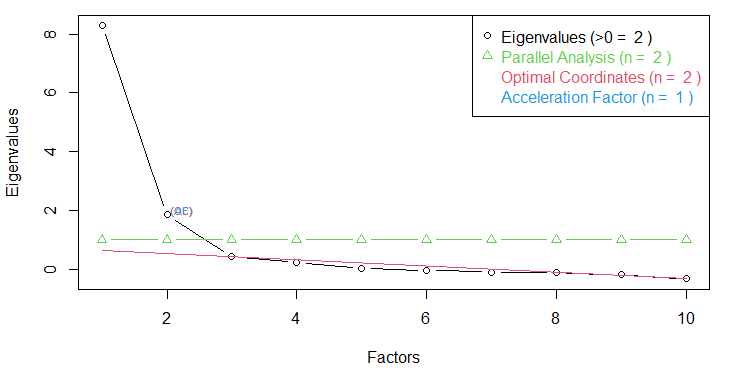


**Study 3**


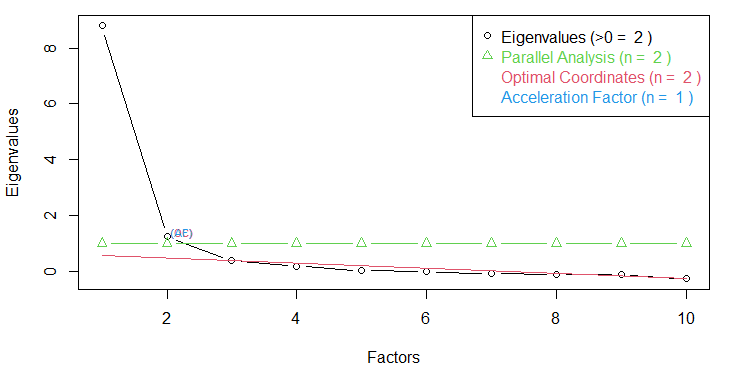


# **References**

Carpenter, S. (2018). Ten Steps in Scale Development and Reporting: A Guide for Researchers. *Communication Methods and Measures*, *12*(1), 25–44. <https://doi.org/10.1080/19312458.2017.1396583>
